# The anatomy of the seed-coat includes diagnostic characters in the subtribe Eugeniinae (Myrteae, Myrtaceae)

**DOI:** 10.3389/fpls.2022.981884

**Published:** 2022-10-05

**Authors:** Patricia Gonçalves Sbais, Nayara Carreira Machado, Karinne Sampaio Valdemarin, Marcela Thadeo, Fiorella Fernanda Mazine, Káthia Socorro Mathias Mourão

**Affiliations:** ^1^Programa de Pós-graduação em Biologia Comparada, Universidade Estadual de Maringá (UEM), Centro de Ciências Biológicas, Maringá, Brazil; ^2^Programa de Pós-graduação em Biologia Vegetal, Instituto de Biologia, Universidade Estadual de Campinas (UNICAMP), Campinas, Brazil; ^3^Departamento de Ciências Ambientais, Universidade Federal de São Carlos (UFSCar), Centro de Ciências e Tecnologias para Sustentabilidade, Sorocaba, Brazil; ^4^Departamento de Biologia, Universidade Estadual de Maringá (UEM), Centro de Ciências Biológicas, Maringá, Brazil

**Keywords:** *Pseudeugenia*, *Racemosae*, *Umbellatae*, *Myrcianthes*, testa, perichalaza, pachychalaza, trait evolution

## Abstract

The subtribe Eugeniinae comprises of two genera, *Eugenia* (ca. 1,100 species) and *Myrcianthes* (ca. 40 species). *Eugenia* is the largest genus of neotropical Myrtaceae and its latest classification proposes 11 sections. This study describes the seed anatomy of forty-one species of Eugeniinae in order to provide possible diagnostic characteristics. Following standard anatomical techniques, flower buds, flowers, and fruits were processed and analyzed using microtome sections and light microscopy. The phylogeny used the regions ITS, *rpl16, psbA-trnH, trnL-rpl32*, and *trnQ-rps16*, following recent studies in the group. Ancestral character reconstruction uncovered that: (1) the ancestral ovule in Eugeniinae was campylotropous (98.9% probability), bitegmic (98.5% probability), and unitegmic ovules arose on more than one lineage independently within *Eugenia*; (2) the pachychalazal seed-coat appeared with a 92% probability of being the ancestral type; (3) non-lignified seed-coat (24,5% probability) and aerenchymatous mesotesta (45.8% probability) are diagnostic characters in *Myrcianthes pungens* (aerenchymatous mesotesta present in the developing seed-coat) and in the species of *E.* sect. *Pseudeugenia* until the species of *E.* sect. *Schizocalomyrtus* and it is the type of seed-coat that predominates in most basal sections on the tree; (4) the partial sclerification (only in the exotesta—exotestal seed-coat) is mainly observed in species of *E.* sect. *Excelsae, E*. sect. *Jossinia* (group X), and *E*. sect. *Racemosae* (22.2% probability); (5) and in the species of the recent lineages of *Eugenia*, with a probability of 27.2%, predominate the exomesotestal or testal construction of the seed-coat [character observed in almost all species analyzed of *E*. sect. *Jossinia* (group Y) and *E*. sect. *Umbellatae*]. A dehiscent fruit is considered as a plesiomorphic state in Myrtaceae; the ancestor of this family had seeds with a completely sclerified testa, and the other testa types described for the current species with dehiscent and indehiscent fruits are simplified versions of this ancestral type. Perhaps, this means that the sclerified layers in the seed-coat have remained in whole or in part as a plesiomorphic condition for taxa with a capsule and bacca. Maintaining the plesiomorphic condition may have represented a selective advantage at some point in the evolutionary history of the family and its groups.

## Introduction

*Eugenia* L. is the largest genus of neotropical Myrtaceae and comprises approximately of 1,100 species (Govaerts et al., 2022). It is the most species-rich genus of angiosperms in Brazil (Proença et al., 2020) and the second richest genus of tree species in the world (Beech et al., 2017).

The subtribe Eugeniinae O. Berg comprises of *Myrcianthes* O. Berg and *Eugenia* L. (Lucas et al., 2007, 2019; Mazine et al., 2014, 2018; Vasconcelos et al., 2017). The last infrageneric classification of *Eugenia* (Mazine et al., 2018) recognizes three major clades classified as subgenera and 11 sections: *Eugenia* subg. *Pseudeugenia* (Mazine and Faria) Mazine and Faria, with *Eugenia* sect. *Pseudeugenia* Mazine and Faria; *Eugenia* subg. *Hexachlamys* (O. Berg) Mattos, with *E.* sect. *Hexachlamys* (O. Berg) Mazine; and *Eugenia* subg. *Eugenia*, with *E.* sect. *Pilothecium* (Kiaersk.) D. Legrand, *E.* sect. *Eugenia*, *E.* sect. *Phyllocalyx* Nied., *E.* sect. *Schizocalomyrtus* (Kausel) Mattos, *E.* sect. *Excelsae* Mazine and E. Lucas, *E.* sect. *Racemosae* O. Berg, *E.* sect. *Umbellatae* O. Berg, *E.* sect. *Speciosae* Bünger and Mazine, and *E.* sect. *Jossinia* (DC.) Nied. (which includes Old World species).

The structure of seeds in Myrtaceae has been taxonomically important since the first classifications of the family (Berg, 1855-1856, 1857, 1858, 1859; Petit, 1908; Gauba and Pryor, 1958; McVaugh, 1968). In addition, the few studies of seed development have revealed that there is a great variation in the seed-coat structure and type of embryo in Myrteae and members of *Eugenia* (Corner, 1976; Van Wyk and Botha, 1984; Moreira-Coneglian, 2007, 2011; Lopes, 2008; Machado, 2014). These studies point to some traits in seed ontogenesis that may show evolutionary trends and be promising diagnostic traits in Myrteae: the ovule types, the variation in the number of integuments and the layers of cells that constitute them; the presence of an obturator; the curvature of the seed; the presence of a pachychalaza or perichalaza; a multiplicative testa; and the presence and position of lignified cells at the end of seed-coat development.

According to Tobe and Raven (1983), all Myrtales have bitegmic ovules, except *Syzygium* Steud. (Myrtaceae). Van Wyk and Botha (1984) stated that *Eugenia* species described in the literature with unitegmic ovules [*E*. *paniculata* (Gaertn.) Britten, *E*. *jambos* L., *E*. *malaccensis* Lour., *E*. *fruticosa* (DC.) Roxb., *E*. *myrtifolia* Jacq.] could be *Syzygium* species. In fact all these species are currently species of *Syzigium* [World Checklist of Selected Plant Families [WCSP], 2022]. A unitegmic ovule was also reported for *Eugenia caryophyllata* Thunb. (Petit, 1908; Tobe and Raven, 1983), which is *Syzygium aromaticum* (L.) Merr. and L. M. Perry. According to Tobe and Raven (1983), it is doubtless that this represents a derived feature, as it generally does in angiosperms (Bouman, 1977) and probably will be found in other genera when the embryology in the family is studied in more detail.

Some of the development studies of Myrteae cited above confirmed that the few *Eugenia* species described have a bitegmic ovule (Van Wyk and Botha, 1984; Moreira-Coneglian, 2007, 2011; Pimentel, 2010; Machado, 2014; Pimentel et al., 2014), but a unitegmic ovule was described for *E*. *uniflora* L. (Lopes, 2008). Pimentel et al. (2014) suggested that the common ancestor of South American and Australasian Myrteae had two integuments protecting the ovules and this character remained present in all members of the group, as in the other Myrtaceae, except for some members of *Syzygium* (tribe Syzygieae).

Corner (1976) proposed the hypothesis that the seeds of the Myrtaceae ancestor had a completely sclerified testa, and the other testa types described for the current species with dehiscent and indehiscent fruits are simplified versions of this ancestral type. The anatomical description of the seed-coat of Eucalyptus L’Hér. species (Petit, 1908; Gauba and Pryor, 1958, 1959, 1961) with capsules and *Decaspermum* J. R. Forst. and G. Forst., *Rhodamnia* Jack, *Rhodomyrtus* DC. (Corner, 1976), *Myrtus* L., *Blepharocalyx* O. Berg., *Psidium* L., *Myrcia* Sol. ex Lindl., *Campomanesia* Ruiz and Pav., *Eugenia* (Petit, 1908; Corner, 1976; Van Wyk and Botha, 1984; Ciccarelli et al., 2005; Moreira-Coneglian, 2007, 2011; Machado, 2014) species having berry-like fruits or like so show that the testa in these genera comprises one or more layers of thick-walled, lignified vs. non-lignified cells. Thus, if Corner’s (1976) hypothesis is correct, simplification occurred in species with dehiscent and indehiscent fruits. In the *Eugenia* species described by Van Wyk and Botha (1984) and Moreira-Coneglian (2011), the testa has varying degrees of sclerification (only exotesta or the whole testa).

Based on the above, the present work describes the seed ontogeny and/or the mature seed-coat of forty-one species the subtribe Eugeniinae not yet described and included in the sections of *Eugenia sensu* Mazine et al. (2018) and *Myrcianthes pungens* (*Myrcianthes* is sister of *Eugenia*) to increase what is known about the seed-coat of the group and answer the following questions: (1) Are there more *Eugenia* species with unitegmic ovule besides *E. uniflora*? (2) Is there variation in the position of the mechanical layers in the mature seed-coat in Eugeniinae? Do these characters have diagnostic importance for the subtribe sections? (3) Can the reconstruction of ancestral characters regarding the seed-coat show evolutionary trends in the subtribe?

## Materials and methods

### Collection and fixation

Flower buds, flowers, and fruits at different development stages from 21 species in subtribe Eugeniinae were collected at the Caiuá Ecological Station, in the municipality of Diamante do Norte, Paraná, Brazil (52° 49′ to 52° 53′ W, 22° 34′ to 22° 37′ S). The vegetation type in this area is semi-deciduous seasonal forest. Voucher material was deposited at HUEM (the State University of Maringá Herbarium) (Supplementary Appendix).

Samples at different developmental stages of the species sampled in the field, taken from one or more herbarium material due to the difficulty of collecting fresh material, are listed in Supplementary Appendix. In all the cases, the samples were identified by specialists. The species in which we were able to carry out the ontogenic study, three specimens of each species, were analyzed to evaluate the variation within the same species that may be due to plasticity and, therefore, without taxonomic value. In 20 species only one specimen was sampled, which were sent by the taxonomist in Eugeniinae and of these 20 species, 18 had mature seed-coat and 2 (*Eugenia cerasiflora* Miq. and *Eugenia dodonaeifolia* Cambess.) had a developing seed-coat.

### Slide preparation

The fresh material was immediately fixed in formaldehyde, acetic acid, and 50% ethyl alcohol (1: 1: 18) and subsequently transferred to and stored in 70% ethanol (Johansen, 1940). Flower buds, flowers, and fruits at different stages of development removed from herbarium were rehydrated for 36 h in a 5% sodium hydroxide solution (Anderson, 1963), thoroughly washed with distilled water, submitted to an increasing ethanol series, and stored in 70% ethanol. For the anatomical study, the material was embedded in Leica historesin after dehydrating it in an ethyl alcohol series (Guerrits and Horobin, 1991).

The material was transversally and longitudinally sectioned at a thickness of about 8 μm with a rotary microtome, stained with toluidine blue in an acetate buffer, pH 4.7 (O’Brien et al., 1964, modified), and mounted in Entelan^®^ synthetic resin.

### Photomicrographic documentation

The slides were analyzed using a Leica DM500 microscope (coupled to a Leica ICC50 camera). The measurements were made with a micrometer using the same optical conditions for each sample.

### Terminology for seed-coat description

The terms used to describe the ovules and seeds are based on Corner (1976).

### Phylogenetic analysis

The phylogeny includes 39 species of Eugeniinae and other five species of Myrteae used as outgroups. The regions ITS, *rpl16, psbA-trnH, trnL-rpl32*, and *trnQ-rps16* were used, following recent studies in the group (Bünger et al., 2016; Mazine et al., 2018). All sequences were obtained on GenBank^1^. Alignments were made on Muscle 3.7 (Edgar, 2004) through CIPRES Science Gateway (Miller et al., 2010). Bayesian inference was conducted using MrBayes 3.2.7a (Ronquist and Huelsenbeck, 2003) through CIPRES Science Gateway. Four independent runs, each with four simultaneous chains, five million generations, and sampled every 1000th generation were applied. Burn-in was set to 25% in MrBayes and effective sample size (ESS) values (> 200) were checked in Tracer v.1.6 (Rambaut and Drummond, 2007). The consensus tree was used as a framework for ancestral character reconstructions. The tree was pruned according with the species data availability for each trait analyzed in this study. Ancestral states were reconstructed with stochastic mapping using the model “equal rates” with the function “make.simmap” available in phytools package (Revell, 2012) implemented in R (R Core Team, 2021). Probabilities were calculated using 10,000 simulations.

## Results

The Supplementary Table 1 summarizes the descriptions of the structural characteristics performed in the present work and in the literature for Eugeniinae and other Myrteae subtribes.

## Seed anatomy in Eugeniinae

### Ovule

#### Curvature and integument number

**FIGURE 1:**
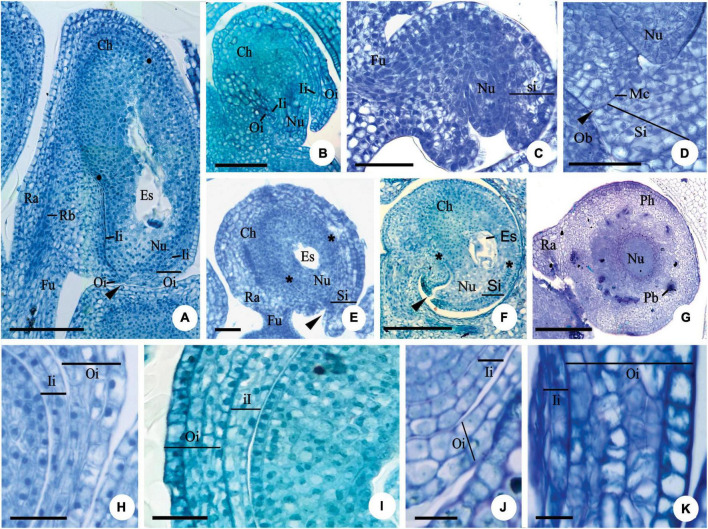
Longitudinal **(A–F,H–I,K)** and transversal **(G,J)** sections of the ovule of flower buds **(B,C)**, flowers at anthesis **(A,E–K)** and post-anthesis **(D)** of *Myrcianthes pungens* **(A,H)**; *Eugenia neoverrucosa* **(B)**; *E. uniflora* **(C–E)**; *E. florida* **(F)**; *Eugenia arenosa* **(G)**; *E. myrciantes* **(I)**; *E. hiemalis* **(J)** and *E. longipedunculata* **(K)**. **(A)** Bitegmic and anatrapous tending to campylotropus ovule and perichalaza (∙) beginning its differentiation. **(B)** Campylotropus ovule evidencing the integuments development. **(C–E)** Development of the campylotropous and unitegmic ovule. Note in photo **(C)** the development of the single integument; in photo **(D)** an obturator in contact to the mycropyle; and in photo **(E)** the pachychalaza (*). **(F)** Campylotropous and unitegmic ovule. Note the extensive pachychalaza and the single integument restricted to the vicinity of the micropyle. **(G)** Ovule showing the ramification of the rapheal bundle in the pachychalazal integument. **(H–K)** Details evidencing the integument layers. Ca, chalaza; Es, embryo sac; Fu, funicle; Ii, inner integument; Mc, micropylar channel; Nu, nucellus; Ob, obturator; Oi, outer integument; Pb, pachychalazal vascular bundle; Pc, pachychalaza; Ra, raphe; Rb, rapheal bundle; Si, single integument; ▶, micropyle. Scale bars: 25 μm **(C,D,H,J,K)**, 50 μm **(A,E)**, 100 μm **(B,I)**, 200 μm **(F,G)**.

For *Myrcianthes pungens* (O. Berg) D. Legrand, the ovule is anatropous, tending to campylotropous (Figure 1A). Most of the studied species of *Eugenia* have a campylotropous ovule (Figures 1B,C,E,F), which is bitegmic (Figures 1A,B,H–K) except *Eugenia arenosa* Mattos, *E*. *dysenterica* DC., *E*. *uniflora* (Figures 1C–E), *E*. *brasiliensis* Lam., *E*. *subterminalis* DC., *E*. *florida* DC (Figure 1F), and *E*. *egensis* DC. The micropylar channel is non-linear in the bitegmic species (Figure 1A); the number of cell layers in the integuments increases in the micropylar region when compared to the number found in the anti-raphe (Figures 1A–F). In *E*. *arenosa*, *E*. *uniflora* (Figure 1E), and *E*. *florida* (Figure 1F), the ovule is pachychalazal and the well-developed pachychalaza is evidenced by the vascular bundles branched throughout the integument, as seen in transverse section (Figure 1G), and by the outer integument restricted to the lower half, as seen in longitudinal section (Figures 1E,F).

#### Integument layers

The number of cell layers in the outer integument (in the median region of the ovule) varies from three to five in *Myrcianthes pungens* (Figure 1H), *Eugenia myrcianthes* Nied. (Figure 1I), *E. langsdorffii* O. Berg, *E. pyriformis* Cambess., *E. expansa* Spring ex Mart., *E involucrata* DC., *E. paracatuana* O. Berg, *E. repanda* O. Berg, *E. gracillima* Kiaersk., *E. hiemalis* (O. Berg) D. Legrand (Figure 1J), *E. neoverrucosa* Sobral, *E. ramboi* D. Legrand, and *E. speciosa* Cambess., and five to seven in *E. arenosa*, *E. dysenterica*, *E. brasiliensis*, *E. longipedunculata* Nied. (Figure 1K), *E. subterminalis*, *E. florida*, and *E. egensis*. The inner integument in the same region has two layers of cells (Figures 1J,K) except *E. myrcianthes* (Figure 1I), *E. langsdorffii*, *E. expansa*, and *E. gracillima*, which have two to three cell layers.

### Seed development

#### Pachychalaza and perichalaza

**FIGURE 2:**
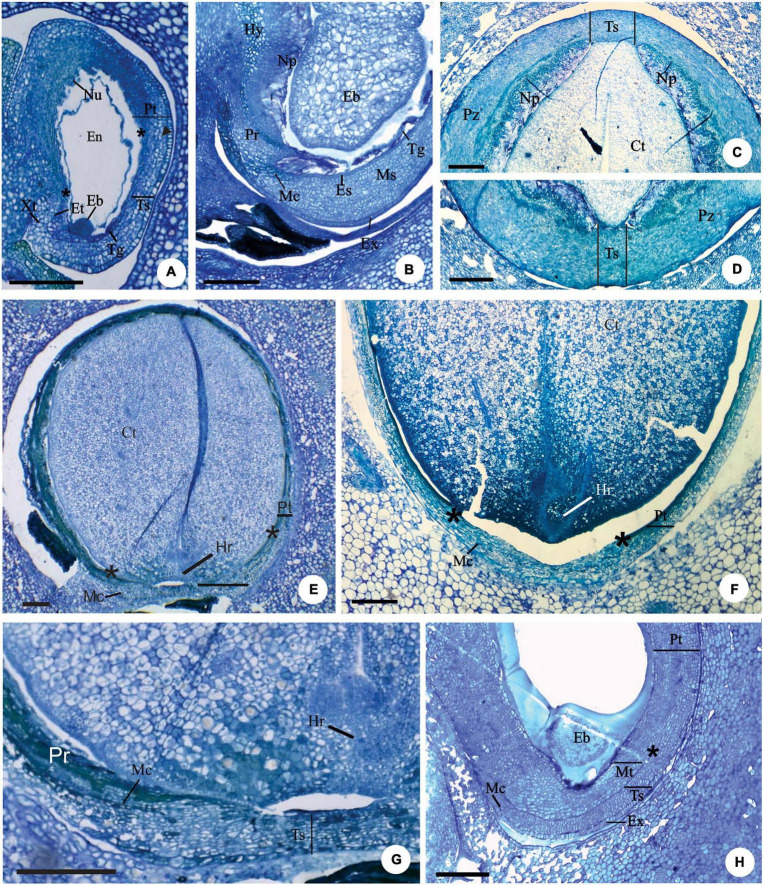
Longitudinal **(A,B,E–H)** and transversal **(C,D)** sections of developing **(A,B,H)** and mature seeds **(C–G)** of *Eugenia longipedunculata* **(A,E,G)**, *E. paracatuana* **(B)**, *E. gracillima* **(C,D)**, *E. involucrata* **(F)** and *E. pyriformis* **(H)**. **(A)** General aspect. Note the non-linear micropylar channel, the nuclear endosperm, the remains of the nucellus, the anticlinal divisions (▶) in the anti-raphe and the limit of the pachychalaza (*). **(B)** Detail evidencing the hypostase, proliferating nucellus, radially elongated exotestal and endotestal cells and mesotesta with divisions in various planes. **(C,D)** Detail showing the perichalazal integument. **(E,F)** General aspect of the markedly campylotropous seed. Note the eugenioid embryo occupying the seed cavity and the limit of the pachychalaza (*). **(G)** Detail of the micropylar region. **(H)** Detail evidencing the multiplicative tegmen and exotesta constituted by radially elongated cells in vicinity of the micropyle. Eb, embryo; Et, endostome; En, nuclear endosperm; Es, endotesta; Ex, exotesta; Hy, hypostase; Hr, hypocotyl-radicle axis; Mc, micropylar channel; Ms, mesotesta; Np, nucellus proliferation; Nu, nucellus; Pr, pre-raphe; Pt, pachychalazal integument; Pz, perichalaza; Tg, tegmen; Tp, multiplicative tegmen; Ts, testa; Xt, exostome. Scale bars = 200 μm **(A,B,E–H)**, 500 μm **(C,D)**.

After fertilization, the chalaza expands, and a pachychalaza develops in species that still lacked this integument in the ovule (Figures 2A,B). In *Myrcianthes pungens* and *Eugenia gracillima*, an extensive perichalaza develops, in which the testa appears as two small lateral bands (Figures 2C,D).

Anticlinal divisions in cells in the antirapheal region (Figure 2A) cause the seed to become markedly campylotropous (Figures 2E,F). Periclinal divisions at the base of the nucellus increase the number of cell layers in this tissue toward the seed cavity; these cells become large and have pectic walls (Figures 2B–D). A hypostasis differentiates at the base of the nucellus and has cells that contain phenolic content (Figure 2B).

In all species, as the embryo develops, the nucellus collapses (Figures 2E–G), the endosperm is nuclear (Figure 2A), and the number of cell layers in the testa, pachychalaza, and perichalaza increases (Figures 2A–D), but remains the same for the tegmen, except in *E. pyriformis*, which has a multiplicative tegmen (Figure 2H).

With the continued development of the seed, the pachychalazal and perichalazal integument constitute the major part of the seed-coat (Figures 2C–H) to all species that developed these integuments. The testa can be seen in the vicinity of the micropyle in the pachychalazal seeds (Figures 2E–H) and in the perichalazal seeds, as well as on both sides of the perichalaza (Figures 2C–D); the tegmen collapses and is no longer visible (Figures 2A–F). The cells of the pachychalaza and perichalaza, especially along the vascular bundles, begin to exhibit phenolic content, mainly in the innermost layers (Figures 2E–G); these cell layers are continuous with the hypostasis cells. With the gradual growth of the embryo, the cells of the nucellus collapse and the endosperm is consumed (Figures 2C–G).

#### Exotesta cells

**FIGURE 3:**
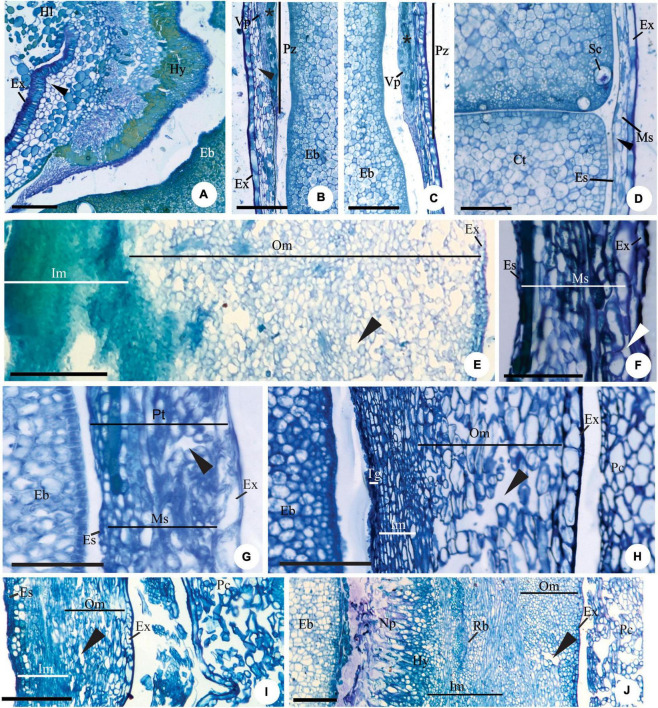
Details of transversal **(B–D,J)** and longitudinal **(A,E–I)** sections showing the seed-coat in the immature **(E–J)** and mature seed **(A–D)** of *Myrcianthes pungens* **(A–D)**, *Eugenia myrcianthes* **(E)**, *E. longipedunculata* **(F)**, *E. uniflora* **(G)**, *E. pyriformis* **(H)**, *E. involucrata* **(I)** and *E. gracillima* **(J)**. **(A)** Hilar region evidencing hypostase with cells of phenolic content and exotesta constituted by macroesclereids (note the aerenchymatic mesotesta). **(B–D)** Seed-coat in perichalaza [pre-raphe – **(B)** and anti-raphe – **(C)]**, and in the testa **(D)**, respectively. Note the thick-walled tabular obliquely elongated exotestal cells, the crushed aerenchymatous mesotesta and the cells of phenolic content in the perichalaza (*). **(E)** Seed-coat in pachychalaza. Note the thin-walled tabular obliquely elongated exotestal cells, the multiplicative mesotesta (outer aerenchymatic), inner and endotesta cells with phenolic content. **(F,G)** Seed-coat in testa and pachychalaza. Note the thin-walled obliquely elongated tabular exotestal cells, the aerenchymatic mesotesta and endotesta crushing. **(H,I)** Seed-coat in testa. Note the obliquely elongated exotestal cells and the aerenchymatic outer mesotesta. **(J)** Seed-coat in rapheal region. Note the remains of the nucellus, the hypostase and the rapheal vascular bundle. Ct, cotyledonous; Eb, embryo; Es, endotesta; Ex, exotesta; Hl, hilum; Hy, hypostase; Im, inner mesotesta; Ms, mesotesta; Np, nucellus proliferation; Om, outer mesotesta; Pc, pericarp; Ph, perichalaza; Pt, pachychalazal integument; Rb, rapheal vascular bundle; Sc, secretory cavity; Ts, testa; Vp, pachychalazal vascular bundle; ▶, intercellular space. Scale bars = 50 μm **(D,F–H)**, 100 μm **(B,C)**, 200 μm **(A,I,J)**, 500 μm **(E)**.

**FIGURE 4:**
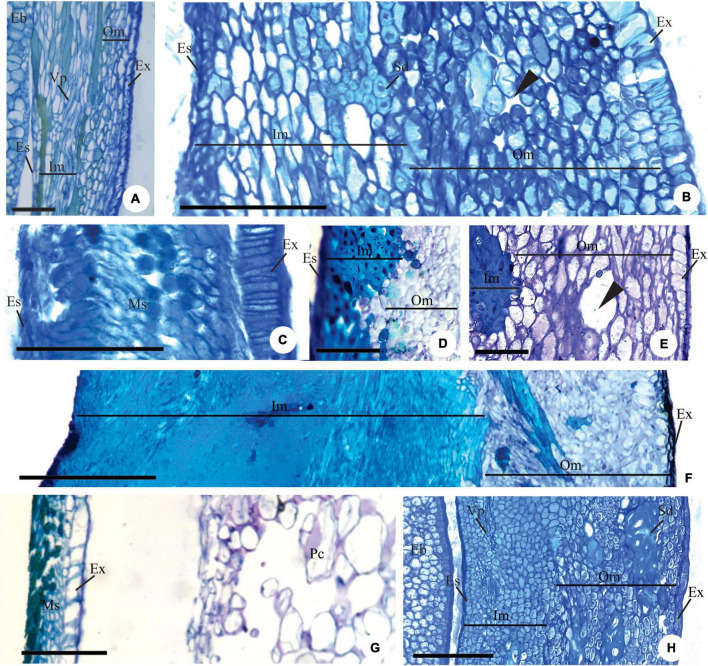
Details of longitudinal sections showing the pachychalazal **(A,B,D–F,H)** and testal **(C,G)** seed-coat in the immature **(A–C)** and mature seed **(D–H)** *Eugenia subterminalis* **(A)**, *E. modesta* **(B)**, *E. goiapabana* **(C)**, *E. arenosa* **(D,E)**, *E. myrcianthes* **(F)**, *E. brasiliensis* **(G)**, and *E. acutata* **(H)**. **(A,B)** Note in photo **(A)** the inner mesotesta with cells stretched longitudinally following the vascular tissue and in photo **(B)** sclereid groups. **(C)** Note that the exotesta cells extend radially and are arranged in a palisade. **(D–F)** Note the sclerified inner mesotesta (inner mesotestal seed). **(G,H)** Note in photo **(G)** the thin-walled exotestal cells and the most cells in the mesotesta and endotesta with phenolic content (non-lignified seed-coat) and in photo **(H)** groups of sclereids in outer mesotesta (outer mesotestal seed). Eb, embryo; Es, endotesta; Ex, exotesta; Im, inner mesotesta; Ms, mesotesta; Om, outer mesotesta; Pc, pericarp; Sd, sclereids; Vp, pachychalazal vascular bundle; ▶, intercellular space. Scale bars = 25 μm **(A)**, 100 μm **(B,C)**, 200 μm **(D–G)**.

Exotesta cells have thin walls, a cuboid or tabular shape in *Myrcianthes pungens* (Figures 3A–D), *Eugena arenosa, E. myrcianthes* (Figure 3E), *E. brasiliensis, E. longipedunculata* (Figure 3F), *E. uniflora* (Figure 3G), *E. pyriformis* (Figure 3H), *E. langsdorffii*, *E. expansa*, *E involucrata* (Figure 3I), *E. repanda*, *E. speciosa, E. dodonaeifolia*, and *E. gracillima* (Figure 3J). In these species, the exotesta cells show tangential-oblique elongation, except in *E. gracillima* (Figure 3J). In *M. pungens*, the exotesta cells close to the funicle are radially elongated and have thickened walls (Figure 3A). In *E. pyriformis*, these cells occur near the micropyle (Figure 2H). The exotesta cells from the micropyle to the edge of the pachychalazal integument extend radially or have a cuboid shape in *E. dysenterica, E. subterminalis* (Figure 4A), *E. florida*, *E. modesta* DC. (Figure 4B), *E. paracatuana* (Figure 2B), *E. cerasifolia, E. goiapabana* Sobral and Mazine (Figure 4C), *E. astringens* Cambess., *E. neoverrucosa*, *E. egensis*, *E. hyemalis*, and *E. ramboi*, and have thin or slightly thickened walls.

#### Mesotesta tissues

Mesotesta is multiplicative, mainly in pachychalaza and perichalaza, and the cells vary in shape and have thin walls in most species (Figures 2A–D,H, 3E–J). It is all aerenchymatous in *Myrcianthes pungens* (Figures 3A–D), *Eugenia brasiliensis, E. uniflora, E. pyriformis, E. involucrata, E. cerasifolia*, and *E. egensis*. In *E. arenosa* (Figure 3E), *E. myrcianthes, E. longipedunculata* (Figure 3F), *E. subterminalis, E. modesta, E. paracatuana*, and *E. gracillima*, only the outer mesotesta is aerenchymatous (Figure 3J). The mesotesta is parenchymatic and consists of isodiametric cells in *E. dysenterica, E. expansa, E. florida, E. repanda, E. speciosa, E. goiapabana, E. astringens, E. neoverrucosa, E. dodonaeifolia, E. hiemalis*, and *E. ramboi.* The inner mesotesta consists of cells that are isodiametrics or stretched in various directions (Figure 4A). In *E. arenosa* and *E. myrcianthes*, the inner mesotesta cells have phenolic content, a thickened wall and begin to differentiate into fibers (Figure 3E).

#### Endotesta cells

The endotesta cells vary in shape (tabular or cuboid) and have thin walls and sometimes phenolic content (Figures 3D,F–I, 4A–C).

### Mature seed

The mature seed is campylotropous and exalbuminous (Figures 2C–G). The embryo is globular in shape and has a short embryonic axis and thick plano-convex cotyledons (Figures 2C–F, 3D).

#### Exotesta cell wall

**FIGURE 5:**
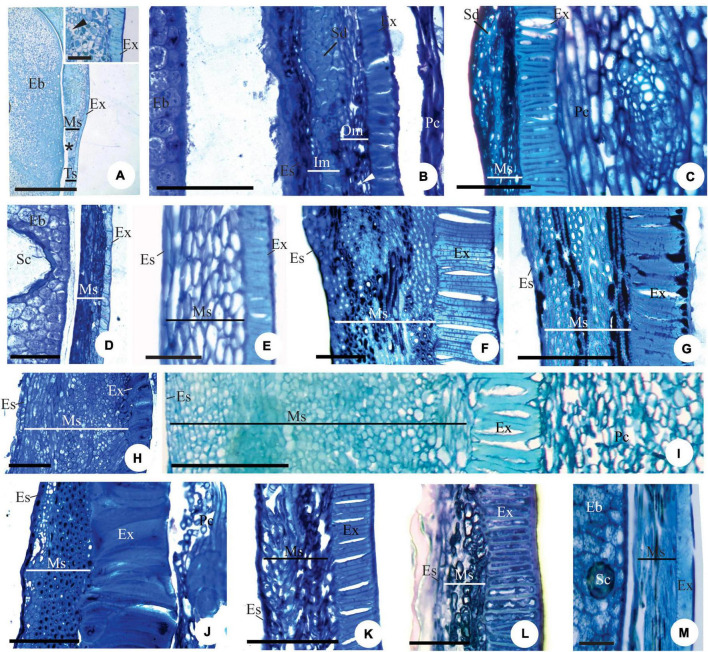
Details of longitudinal sections of mature seed showing the pachychalazal **(A–C,F–J)** and testal **(D,K–M)** seed-coat of *Eugenia subterminalis* **(A)**, *E. excelsa* **(B)**, *E. florida* **(C)**, *E. modesta* **(D)**, *E. paracauana* **(E)**, *E. bahiensis* **(F)**, *E. hirta* **(G)**, *E. leptoclada* **(H)**, *E. neoverrucosa* **(I)**, *E. flavescens* **(J)**, *E. batingabranca* **(K)**, *E. egensis* **(L)**, and *E. hiemalis* **(M)**. Note the exotesta constituted by a palisade of radially elongated lignified macroesclereids **(A–C,E–L)**. In photos **(D,M)** these cells are cuboidal in shape. Note the exotestal seed in photos **(A,D,E,H,I,K,L)**, exomesotestal in photos **(B,C)** and testal in photos **(F,G)**. Eb, embryo; Es, endotesta; Ex, exotesta; Im, inner mesotesta; Ms, mesotesta; Om, outer mesotesta; Pc, pericarp; Vp, pachychalazal vascular bundle; ▶, intercellular space. Scale bars = 25 μm **(M)**, 50 μm **(G,L)**, 100 μm **(B–D,F,H,J,K)**, 200 μm **(I)**, 400 μm **(A)**.

There is variation in the thickening of the cell walls of the mature seed-coat (Figures 3A–D, 4D–H, 5A–M). Exotesta cells have thin walls in *Eugenia arenosa* (Figure 4E), *E. klotzschiana* O. Berg, *E. myrcianthes* (Figure 4F), *E. brasilensis* (Figure 4G), *E. longipedunculata* (Figure 2G), *E. uniflora*, *E. langsdorffii, E. supraaxillaris* Spring, *E. involucrata* (Figure 2F), *E. acutata* Miq. (Figure 4H), *E. arvensis* Vell., *E. patens* Poir., *E. repanda*, *E. speciosa*, and *E. gracillima*. *Myrcianthes pungens* has non-lignified cells with thick walls, but the cells close to the hilum differentiate into radially elongated macrosclereids with lignified walls (Figures 3A–D). In *E. pyriformis*, these macroesclereids differentiate from the end of the pachycalazal integument toward the micropyle. In *E. subterminalis* (Figure 5A), *E. excelsa* O. Berg (Figure 5B), *E. florida* (Figure 5C), *E. modesta* (Figure 5D), *E. paracatuana* (Figure 5E), *E. bahiensis* DC. (Figure 5F), *E. hirta* O. Berg (Figure 5G), *E. subavenia* O. Berg, *E. stictopetala* Mart. ex DC., *E. pluriflora* DC., *E. leptoclada* O. Berg (Figure 5H), *E. neoverrucosa* (Figure 5I), *E. flavescens* DC. (Figure 5J), *E. batingabranca* Sobral (Figure 5K), *E. egensis* (Figure 5L), *E. hiemalis* (Figure 5M), *E. mosenii* (Kausel) Sobral, and *E. ramboi*, the exotesta comprises thick-walled and lignified cells that vary from cuboid (Figures 5D,M) to radially elongated macrosclereids (Figures 5A–C,E–L).

#### Mesotesta tissues

In *Eugenia myrcianthes, E. arenosa*, and *E. klotzschiana*, the outermost layers of the mesotesta are aerenchymatous and the inner mesotesta consist of fibers extending in different directions, with thickened and lignified walls that make the seed-coat very hard in the first two species (Figures 4D–F). The aerenchymatous or parenchymatic tissue is visible or may be crushed (Figures 3A–D, 4D–H, 5A–E,H,I,K,L). In *E. leptoclada* and *E. batingabranca*, all mesotesta is constituted by non-lignified fiber-like and isodiametric cells (Figures 5H,K). In *E. bahiensis, E. hirta, E. stictopetala, E. flavescens, E. hiemalis*, and *E. ramboi*, these cells are lignified (Figures 5F,G,J,M). In *E. pluriflora* and *E. egensis*, lignified fiber-like cells occur in outer mesotesta. The inner mesotesta is constituted by non-lignified (*E. subterminalis* and *E. paracatuana*) (Figures 5A,E) or lignified (*E. speciosa, E. gracillima, E. subavenia*, and *E. mosenii*) isodiametric or fiber-like cells. In *E. acutata* (Figure 4H), *E. excelsa* (Figure 5B), *E. arvensis*, *E. modesta*, and *E. florida*, groups of sclereids appear in the inner mesotesta in pachychalazal seed-coat. In *E. langsdorffii* and *E. supraaxillaris*, there are many groups of sclereids throughout the parenchymatic mesotesta. *E. neoverrucosa* has a mesotesta with many layers of isodiametric cells and a compact arrangement (Figure 5I).

#### Endotesta cells

The endotesta cells are crushed in most species, but it consists of fiber-like cells strongly lignified in *E. arenosa.* In *E. klotzschiana*, these cells are cuboid in shape and lignified. The cuboid endotestal cells in *E. involucrata* have thin walls, but in *E. acutata* and *E. arvensis*, the walls are thick, but not-lignified.

#### Tegmen

As mentioned, the tegmen in most species becomes compressed as the seed develops, but in *E. pyriformis*, it can be observed until the seed matures. In addition, the pachychalazal and perichalazal integument differs from the testa itself because it has more cell layers and the presence of vascular bundles (Figures 2C,D, 3B,C, 4D–H, 5A–M).

#### Embryo tissues

In the embryo, which is covered by the protoderm, procambial bundles that branch-off toward the cotyledons and secretory cavities are present in the fundamental meristem (Figures 2E,F). The cells of the fundamental meristem have a starch reserve.

**FIGURE 6:**
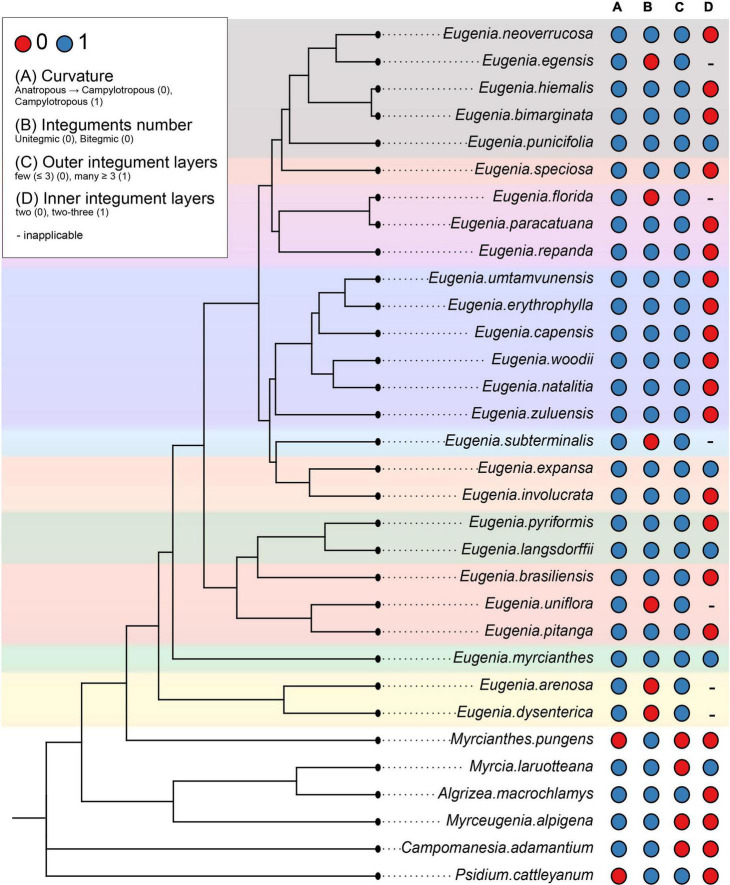
Eugeniinae phylogeny with species character states of ovule: curvature **(A)**, integuments number **(B)**, outer integument layers **(C)**, and inner integument layers **(D)** (_
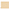
_ *Eugenia* sect. *Pseudeugenia*;_
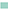
_ *E.* sect. *Hexachlamys*;_
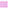
_ *E.* sect. *Eugenia*;_
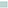
_ *E.* sect. *Pilothecium*;_
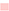
_ *E.* sect. *Phyllocalyx*;_
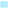
_ *E.* sect. *Schizocalomyrtus*;_
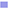
_ *E.* sect. *Jossinia*;_
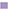
_ *E.* sect. *Racemosae*;_
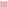
_ *E.* sect. *Speciosae*;_
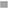
_ *E.* sect. *Umbellatae*).

**FIGURE 7:**
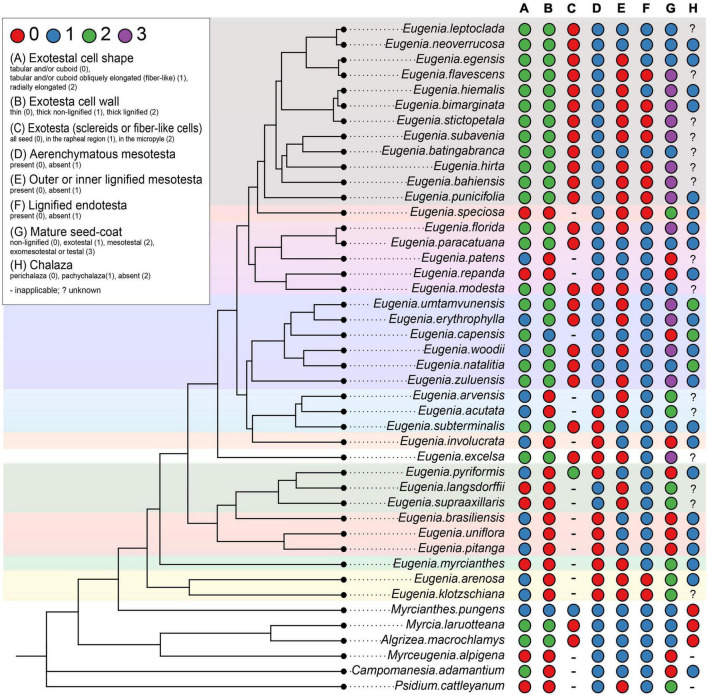
Eugeniinae phylogeny with species character states of mature seed: exotestal cell shape **(A)**, exotesta cell wall **(B)**, exotesta surface **(C)**, mesotesta **(D)**, outer and/or inner mesotesta **(E)**, endotesta lignification **(F)**, mature seed coat **(G)**, and chalaza **(H)** (_
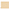
_ *Eugenia* sect. *Pseudeugenia*;_
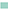
_ *E.* sect. *Hexachlamys*;_
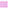
_ *E.* sect. *Eugenia*;_
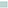
_ *E.* sect. *Pilothecium*;_
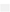
_ *E.* sect. *Excelsae*;_
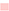
_ *E.* sect. *Phyllocalyx*;_
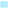
_ *E.* sect. *Schizocalomyrtus*;_
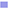
_ *E.* sect. *Jossinia*;_
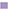
_ *E.* sect. *Racemosae*;_
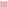
_ *E.* sect. *Speciosae*;_
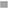
_ *E.* sect. *Umbellatae*).

The scores for potentially 12 informative anatomical character states of the ovule and the mature seed of Subtribe Eugeniinae and other Myrteae subtribes (original data and taken from the literature) are in Supplementary Table 2. Figures 6, 7 summarize the Eugeniinae phylogeny with species character states of ovule and mature seed-coat.

## Ancestral state reconstructions

### Ovule

#### Curvature and integument number

Ancestral state reconstructions of ovule curvature provided that the possible phenotype of Eugeniinae ancestral was campylotropous (98.9% probability of campylotropous; 1.1% probability of anatropous tending to campylotropous; Supplementary Figure 1). Ovule curvature was reconstructed as a conserved trait since 2.16 changes between states on average were estimated. For the integuments number, the Eugeniinae ancestral was reconstructed as bitegmic (98.5% probability of bitegmic; 1.5% probability of unitegmic), with 7.2694 changes between states on average estimated and 5.9319 reversals on average; unitegmic ovules arose on more than one linage independently within *Eugenia* (Supplementary Figure 2).

#### Integument layers

The outer integument phenotype of Eugeniinae ancestral was reconstructed with a higher probability having more than three layers (70.4% probability of more than 3 layers; 29.6% probability of less than 3 layers), with 4.443 changes between states on average estimated and 2.7078 reversals on average. The number of outer integument layers was reconstructed as a conserved trait among *Eugenia* sections (Supplementary Figure 3). For the inner integument, Eugeniinae ancestral probably had two layers (93.11% probability of two layers; 6.89% probability of two to three layers), with 8.5558 changes between states on average and 2.5911 reversals on average; two to three layers arose on more than one linage independently within *Eugenia* (Supplementary Figure 4).

### Mature seed

#### Pachychalaza and perichalaza

The chalaza of Eugeniinae ancestral was reconstructed with a higher probability to be pachychalaza (81.3% probability of pachychalaza; 17.4% probability of perichalaza; 1.3% probability of being absent; Supplementary Figure 5), with 6.2365 changes between states on average.

#### Exotesta cell shape

The exotesta cell shape of Eugeniinae ancestral had a probability of 48.6% to be tabular and/or cuboid, 30.8% to be tabular and/or cuboid obliquely elongated (fiber-like), and 20.5% to be radially elongated (Supplementary Figure 6), with 29.2626 changes between states on average. Regarding the exotesta recovery, Eugeniinae ancestral had a probability of 91.3% of having sclereids or fiber-like cells in all seeds (6% probability of having just in the rapheal region; 2.7% probability of having in the micropyle; Supplementary Figure 7). Exotesta recovery was reconstructed as a conserved trait since 2.3177 changes between states on average were estimated.

#### Exotesta cell wall

For the exotesta cell wall, Eugeniinae ancestral was reconstructed with a higher probability of being thin (93.1% probability to be thin; 3.3% probability to be thick and non-lignified; 3.6% probability to be thick and lignified; Supplementary Figure 8), with 13.4 changes between states on average.

#### Mesotesta tissues

The mesotesta tissues of Eugeniinae ancestral had a 54.2% probability of not being aerenchymatous (45.8% probability of being aerenchymatous; Supplementary Figure 9), with 17.1217 changes between states on average. Also, the outer or inner mesotesta tissues of Eugeniinae ancestral were reconstructed with 50.3% probability of being lignified and 49.7% probability of being non-lignified (Supplementary Figure 10). The outer or inner mesotesta tissues were not recovered as a conserved trait among *Eugenia* sections since 106.3287 changes between states on average were estimated.

#### Endotesta cell

Endotesta cell was recovered with 97% probability of being non-lignified in Eugeniinae ancestral, with 9.4516 changes between states on average; lignified endotesta arose on more than one linage independently within *Eugenia* (Supplementary Figure 11).

#### Mature seed-coat

The seed-coat of Eugeniinae ancestral had a probability of 27.2% of being exomesotestal or testal, 26.1% mesotesal, 24.5% non-lignified, and 22.2% exotestal; with 60.8347 changes between states on average (Supplementary Figure 12).

## Discussion

The results of the present study and information in the literature (Supplementary Tables 1, 2) reveal some characteristics of the seeds that may provide a better understanding by Eugeniinae and Myrteae.

### Ovule curvature

The curvature of the ovule, observed in *Eugenia* species in the present study, is in accordance with the descriptions of Van Wyk and Botha (1984) for this genus as hemi-campylotropous, tending to ana-campylotropous. Variations in ovule curvature are also found in the descriptions of Myrtaceae by Corner (1976) (campylotropous ovule) and Nic Lughadha and Proença (1996) (anatropous ovule), as well as at higher hierarchical levels, such as those found in Tobe and Raven (1983) for Myrtoideae (anatropous ovules) and Endress (2010) for Myrtales (campylotropous ovule). All these results demonstrate the need to increase embryological studies in Myrtaceae; so this character can be safely used in phylogenetic discussions, despite the ancestral state reconstructions of ovule curvature provided that the possible phenotype of Eugeniinae ancestral was campylotropous (98.9% probability).

### Integument layers

A unitegmic ovule in *Eugenia* had already been described for *E. uniflora* by Lopes (2008), and the present study found new records of this ovule type. Wilson (2011) stated that the bitegmic is the most common ovule type in Myrtaceae, and Pimentel et al. (2014) assumed that the ancestor of Myrteae from South America and Australasia had a bitegmic ovule, and this character state remained present in all species, being a synapomorphy for the group. According to Doyle and Endress (2000) and Endress and Doyle (2009), the ancestor of angiosperms probably had two integuments. In this respect, the loss of an integument during the evolution of Myrtaceae is a derived state, as interpreted by Tobe and Raven (1983) and Wilson (2011). In this study, the Eugeniinae ancestral was reconstructed as bitegmic (98.5% probability) and unitegmic ovules arose on more than one lineage independently within *Eugenia*.

The outer integument in Eugeniinae had a higher probability of having more than three layers (70.4% probability), and it showed as a conserved trait among *Eugenia* sections. Two to three layers arose on more than one linage independently within *Eugenia*. The presence of two layers in the inner integument found in most species of subtribe Eugeniinae in this study showed 93.11% probability to be the ancestral state in Eugeniinae, and it was described for Myrteae by Van Wyk and Botha (1984), Moreira-Coneglian (2007, 2011), Pescador et al. (2009), and Machado (2014), for Myrtaceae by Corner (1976), and for Myrtales by Tobe and Raven (1983) and Endress (2010). The proliferative inner integument in *E. pyriformis*, discernable until the mature seed, is a new record for the family. Studies of ovule ontogeny in more species of subtribe Eugeniinae may reveal transitional states: species showing a multiplicative inner integument; species with different degrees of reduction in the number of layers of the inner integument; and species with only one integument, but whose common ancestor had a bitegmic ovule, a plesiomorphic condition to all Myrtales and angiosperms according to Doyle and Endress (2000) and Endress and Doyle (2009), condition found to have 98.5% probability of ancestry in Eugeniinae.

### Micropyle

The variation in the micropyle structure reported in the literature reinforces the importance of describing the ontogeny of the ovules for a representative number of species before using related characters in phylogenetic discussions. All *Eugenia* species have a micropylar channel consisting of the endostome and exostome; although, Van Wyk and Botha (1984) report that the micropyle is formed by the endostome only in *E. woodii* Dümmer. Also, for Myrtaceae in general, Corner (1976) observed that the micropyle is formed exclusively by the exostome and, according to Machado (2014), this can be explained by the fact that the outer integument develops faster than the inner integument, as observed by this author for the described species (including *E. pitanga*), but at the end of the ovule development, the micropylar channel is formed by both integuments. This type of development was observed in the present study and was also reported by Moreira-Coneglian (2007, 2011).

The non-linear micropyle is a highly variable character between species of different sections and within the same section, as seen in the present study, which makes its use in phylogenetic discussions difficult. This character was also reported for other species of Myrteae that belong to subtribes Pimentinae and Myrciinae (Moreira-Coneglian, 2007; Pescador et al., 2009; Machado, 2014). Endress (2011) reported that in campylotropous ovules, the anti-raphe develops strongly, so that the outer integument becomes long and overlaps the inner integument, which gives rise to a “zig-zag” appearance. This greater development of the outer integument was also observed in the present study. However, as mentioned, descriptions of more species that include details about the ontogeny of ovules could reveal if this character is phylogenetically useful.

### Pachychalaza and perichalaza

The development of a pachychalaza is associated with species with large recalcitrant embryos, as observed here for most species of *Eugenia*, which is a plesiomorphic character that was described for the genus by Von Teichman and Van Wyk (1991). In character reconstruction for Eugeniinae, this character showed a high probability of being ancestral in the subtribe (81.3%).

According to Moreira-Coneglian (2011), large, recalcitrant seeds with a pachychalaza, as in *Eugenia*, may be related to desiccation tolerance. Moreira-Coneglian (2011) based this statement on desiccation tolerance studies of seeds of *E*. *stipitata* (Gentil and Ferreira, 1999), *E*. *pyriformis* (Andrade and Ferreira, 2000), *E. calycina* Cambess (currently synonymous with *E*. *involucrata* DC.) (Bülow et al., 1994), and *E. involucrata* (Maluf et al., 2003). Further studies on the structure of the ovaries and seeds are needed to correlate characters with the variations in environmental conditions of the ecosystems where the species have been selected throughout their evolutionary histories.

According to Corner (1976), the seeds of several species of *Eugenia* are unitegmic and the seed-coat may be pachychalazal but some species described by the author as *Eugenia* are currently circumscribed in *Syzigium*. Partly pachychalaza is a particularly well-developed structure in the members of *Eugenia* group Y, an informal group of species confined to South Africa (Van Wyk and Botha, 1984). A pachychalaza also occurs in *Eugenia punicifolia, E*. *bimarginata* and *E*. *aurata* (Moreira-Coneglian, 2007, 2011), *E*. *pitanga* Kiaersk., *Campomanesia adamantium* (Cambess.) O. Berg (Pimentinae), *Myrcia multiflora* DC. (Myrciinae), and *Myrciaria delicatula* (DC.) O. Berg (Pliniinae) (Machado, 2014).

In *Myrcianthes pungens* and *Eugenia gracillima*, a perichalaza develops, as reported by Moreira-Coneglian (2007, 2011) for species of *Myrcia* DC. (Myrciinae) and *Blepharocalyx salicifolius* (Kunth) O. Berg (Blepharocalycinae) and by Galan (2020) for species of *Siphoneugena* O. Berg, *Neomitranthes* D. Legrand, *Plinia* L., *Myrciaria* O. Berg, and *Algrizea* Proença and NicLugh. (Pliniinae). Although the presence of a perichalaza in Myrteae has only been reported for some species, it is necessary to carry out the reconstruction of this character in the tribe to verify what is the probability of this character having preceded the appearance of pachychalaza, since in Eugeniinae, the probability was low (17.4%) in the reconstruction analysis, as well as the absence of pachychalaza and perichalaza (1.3% probability). Another aspect to consider is whether this character is related to the greater or lesser recalcitrance of seeds selected by environmental pressure throughout the evolutionary history of the species.

### Seed-coat tissues × diagnostic features for Eugeniinae sections

The results of our ontogenic study of the seed-coat of species of subtribe Eugeniinae show variations in cell shape, spacing, and lignification of cell layers.

In *Myrcianthes pungens* and in species of *Eugenia* sect. *Pseudeugenia*, *E.* sect. *Hexachlamys*, *E.* sect. *Eugenia*, *E.* sect. *Pilothecium*, and *E.* sect. *Phyllocalyx*, there was uniformity regarding the occurrence of cells in the exotesta with tabular-cuboid format, obliquely elongated (fiber-like) or not, feature that showed a 48.6% probability of being the ancestral type in the reconstruction analysis, followed by a 30.8% probability of being obliquely elongated. Seeds with non-lignified seed-coat, with lignified inner mesotesta or fully mesotesta, occur in *M. pungens* and in the species of *E.* sect. *Pseudeugenia*, *E.* sect. *Hexachlamys*, *E.* sect. *Eugenia*, *E.* sect. *Pilothecium, E.* sect. *Phyllocalyx*, and *E.* sect. *Schizocalomyrtus*, although in *M. pungens*, the cells are thick-walled. Seeds with this construction were also observed in *E. patens* and *E. repanda* (*E.* sect. *Racemosae*), *E. speciosa* (*E.* sect. *Speciosae*), and *E. gracillima* (*E.* sect. *Umbellatae*). The probability of this type of construction in the seed-coat being ancestral to the Eugeniinae subtribe was high (93.1% thin-walled, non-lignified) with 3.3% probability to be thick and non-lignified. It is important to note that the exotesta of cells with non-lignified walls is mostly associated with an aerenchymatous mesotesta, a character that showed a 54.2% probability of being ancestral in the subtribe.

However, in spite of seeds with non-lignified seed-coat were also observed in species of *Eugenia* sect. *Racemosae* (*E. patens* and *E. repanda*), *E.* sect. *Speciosae* (*E. speciosa*) and *E.* sect. *Umbellatae* (*E. gracillima*) and also in *E.* sect. *Jossinia*, whose species were described by Van Wyk and Botha (1984), there is a predominance of exotestal (only the exotesta is lignified), exomesotestal (the exotesta and mesotesta are lignified), and testal seeds (all testa is lignified) in these sections, as well as *E*. sect. *Schizocalomyrtus* (*E. subterminalis*). In these sections, these seeds are associated with a palisade of radially elongated macrosclereids (20.5% probability of being the ancestral in Eugeniinae), although lignified fiber-like cells or cuboid sclereids also occur.

Van Wyk and Botha (1984) stated that exotestal seeds are most frequent in *Eugenia* group X and exomesotestal most frequent in *Eugenia* group Y. In the present study, it can be observed that there is a predominance of exomesotestal to completely testal seeds in the species of *E.* section *Umbellatae*, as also found by Moreira-Coneglian (2007, 2011) in *E. aurata* O. Berg, *E. bimarginata* DC., and *E. punicifolia* (Kunth) DC. (all circumscribed in *E.* section *Umbellatae*). According to Mazine et al. (2018), this section contains approximately 500 species, or around half of the species in the genus.

Exomesotestal or testal seeds in species of *E*. sect. *Umbellatae* and *E*. sect. *Jossinia* (group Y) are probably a diagnostic feature with a probability of 27.2% of being the ancestral seed-coat type of Eugeniinae. The partial sclerification (only in the exotesta), observed in species of *E*. sect. *Schizocalomyrtus*, *E*. sect. *Excelsae, E*. sect. *Jossinia* (group X), and *E*. sect. *Racemosae* (22.2% probability in Eugeniinae ancestral) and the non-lignified seed-coat (parenchymatic or aerenchymatous mesotesta) or the mesotestal seeds (26.1% mesotesal, 24.5% non-lignified in Eugeniinae ancestral), are also probably diagnostic features. These are good characters that should be explored, including how they are related to the diversification of subtribe Eugeniinae.

### Fruit opening × lignified seed-coat

The dehiscent fruit is considered a plesiomorphic state in Myrtaceae (Wilson et al., 2001) and Corner (1976) hypothesized that the ancestor of this family had seeds with a completely sclerified testa and the other testa types are simplified versions of this type. Thus, this simplification occurred in species with dehiscent or indehiscent fruits, as can be seen in the seed descriptions of *Eucalyptus* (Petit, 1908; Gauba and Pryor, 1958, 1959, 1961) with capsules and *Decaspermum*, *Rhodamnia*, *Rhodomyrtus* (Corner, 1976) *Myrtus*, *Blepharocalyx*, *Psidium*, *Myrcia*, *Campomanesia*, *Eugenia* (Petit, 1908; Narayanaswasm and Roy, 1960; Corner, 1976; Van Wyk and Botha, 1984; Ciccarelli et al., 2005; Moreira-Coneglian, 2007, 2011; Machado, 2014) having berry-like fruits or like so. In these genera, the testa consists of one or more layers of thick-walled, lignified vs. non-lignified cells.

Perhaps, this means that the sclerified layers in the seed-coat have remained in whole or in part as a plesiomorphic condition for taxa with capsule or having berry-like fruits or like so. Maintaining the plesiomorphic condition may have represented a selective advantage at some point in the evolutionary history of the family and its groups.

Vasconcelos et al. (2017) stated that from a dry biome origin, the common ancestor of modern *Eugenia* and *Myrcianthes* took advantage of the extensive humid forests in the Oligocene and the beginning of the Miocene, where *E*. subg. *Eugenia* subsequently underwent high levels of rapid speciation. The predominance of exomesotestal or testal seeds in species of *E*. sect. *Umbellatae* and *E*. sect. *Jossinia* (group Y) may be the plesiomorphic condition that persisted in these groups. It is interesting to note that in *M. pungens*, the fruit is like a drupe, which is a recently recorded trait for this genus (Sbais et al., 2022), and that the seed-coat is mostly made up of slightly thick-walled cells. Results from more comprehensive studies of the fruits and seeds of Myrtaceae that focus on these characters, as well as studies that combine geographic distribution and dispersion, might support these hypotheses.

## Conclusion

Some characteristics regarding seed ontogenesis of species of subtribe Eugeniinae deserve to be highlighted, such as the following: (1) The ancestral ovule in Eugeniinae was campylotropous (98.9% probability); (2) the new records of unitegmic ovules in *E*. *arenosa*, *E*. *dysenterica*, *E. brasiliensis*, *E. uniflora, E. subterminalis, E*. *florida*, and *E. egensis* proved to be a feature that arose on more than one lineage independently within *Eugenia*; (3) the pachychalazal seed-coat appeared with a 92% probability of being the ancestral type; (4) the exotesta of cells with non-lignified walls, mostly associated with an aerenchymatous mesotesta, is a character that showed a 54.2% probability of being ancestral in the subtribe; and it is the type of seed-coat that predominates in the most basal sections on the tree (*E.* sect. *Pseudeugenia, E.* sect. *Hexachlamys, E.* sect. *Eugenia* and *E.* sect. *Pilothecium*) and also occurs in *Myrcianthes pungens* (aerenchymatous mesotesta present in the developing seed-coat); this type of construction is most frequently observed until the species of *E.* sect. *Schizocalomyrtus;* (5) the partial sclerification (only in the exotesta—exotestal seed-coat) mainly occurs in species of *E.* sect. *Excelsae, E*. sect. *Jossinia* (group X), and *E*. sect. *Racemosae* (22.2% probability); (6) and in the species of the recent lineages of *Eugenia*, with a probability of 27.2%, occurs the exomesotestal (exotesta and mesotesta lignified) or testal (all testa is lignified) construction of the seed-coat [character observed in almost all species analyzed of *E*. sect. *Jossinia* (group Y) and *E*. sect. *Umbellatae*], considered the plesiomorphic state for the family Myrtaceae.

## Data availability statement

The original contributions presented in the study are included in the article/Supplementary material, further inquiries can be directed to the corresponding author/s.

## Author contributions

PS: investigation, writing—original draft, and visualization. NM and KV: investigation and visualization. FM and MT: conceptualization and writing—review and editing. KM: conceptualization, resources, writing—review and editing, supervision, and project administration. All authors contributed to the article and approved the submitted version.

## Footnotes

1
https://www.ncbi.nlm.nih.gov/genbank/
