# Supplementary material for: The anatomy of the seed-coat includes diagnostic characters in the subtribe Eugeniinae (Myrteae, Myrtaceae)
Source: Front Plant Sci. 2022 Oct 5;13:981884. doi: 10.3389/fpls.2022.981884 (PMC9580042; doi:10.3389/fpls.2022.981884)
Supplement: Supplementary file 1 [file Data_Sheet_1.pdf]

Supplementary Appendix

Voucher information: *Myrcianthes pungens* (O.Berg) D.Legrand: Estação Ecológica de Caiuá, trilha do Mico, Diamante do Norte, PR, Brasil. Col.: M.B Romagnolo nº 3193. Det.: M.B Romagnolo (HUEM 21,410) / Estação Ecológica de Caiuá, estrada da água mole, Diamante do Norte, Paraná, Brasil. Col.: L.S Souza, s/n. Det.: M.B Romagnolo (HUEM 16,384) / Estação Ecológica de Caiuá, Sede, Diamante do Norte, Paraná, Brasil. Col.: G.O Landgraf et al. nº 36. Det.: M.B Romagnolo (HUEM 22,762). *Eugenia arenosa* Mattos: Rod. MS-345, Bonito - Anastácio, próximo a Fazenda Serra Verde, Bonito, Mato Grosso do Sul, Brasil. Col.: G. Hatschbach et al. nº 76146. Det.: M. Sobral (MBM 287,767) / Itararé, São Paulo, Brasil. Col.: F.F Mazine et al., nº 1043. Det.: M. Sobral (MBM 297301). *Eugenia klotzschiana* O. Berg: Reserva Ecológica do IBGE, Brasília, Distrito Federal, Brasil. Col.: V.C. Souza nº 39676. Det.: Flores, T.B. & Colleta, T.D. (ESA 137,729). *Eugenia dysenterica* (Mart.) DC.: Rodovia BR-135, próximo ao km 392, Montes Claros, Minas Gerais, Brasil. Col.: G. Hatschbach nº 73662. Det.: M. Sobral (MBM 272,366) / Serra das Araras, Alto Paraguai, Mato Grosso, Brasil. Col.: B. Dubs nº 1697. Det.: M.L. Kawasaki (MBM 222,097) / Fazenda Ipameri, Corumbá, Mato Grosso do Sul, Brasil. Col.: G. Hatschbach nº 60923. Det.: H.S. Silva (MBM 167,951). *Eugenia myrcianthes* Nied.: Rio Paraná, faixa marginal ao rio, entorno da área de reflorestamento, São Pedro do Paraná, Paraná, Brasil. Col.: Mª. C Souza et al. nº 2122. Det.: G.S. Rosa (HUEM 17,689) / Rio Paraná margem esquerda, Marilena, Paraná, Brasil. Col.: M.B Romagnolo nº 570. Det.: M.B Romagnolo (HUEM 9,362) / Fazenda Santa Isabel, Presidente Castelo Branco, Paraná, Brasil. Col.: M.B Romagnolo nº 3372. Det.: M.B. Romagnolo (HUEM 24,165). *Eugenia brasiliensis* Lam.: Horto de Plantas Medicinais Irenice Silva, UEM, Maringá, Paraná, Brasil. Col.: G. Hatschbach nº 13127. Det.: M.B. Romagnolo (HUEM 4,761) / Horto de Plantas Medicinais Irenice Silva, UEM, Maringá, Paraná, Brasil. Col.: I.Silva s/n. Det.: G.M. Barroso (HUEM 2,435). *Eugenia longipedunculata* Nied.: Estação Ecológica do Caiuá, estrada do ribeirão Conceição, Diamante do Norte, Paraná, Brasil. Col.: M.B. Romagnolo nº 3391. Det.: M.B Romagnolo (HUEM 25,099) / Bosque Municipal de Paranavaí, Paranavaí, Paraná, Brasil. Col.: M. B. Romagnolo nº 3115. Det.: M.B Romagnolo (HUEM 20,495) / Estação Ecológica do Caiuá, Diamante do Norte, Paraná, Brasil. Col.: G.O. Landgraf nº 33. Det.: M.B Romagnolo (HUEM 22,518). *Eugenia uniflora* L.: Estação Ecológica do Caiuá, Mata do Ribeirão Conceição. Diamante do Norte, Paraná, Brasil. Col.: F. Barragam nº 18. Det.: M.B. Romagnolo (HUEM 16,366) / Estação Ecológica do Caiuá, Trilha das araras. Diamante do Norte, Paraná, Brasil. Col.: G.O. Landgraf et al. nº 44. Det.: M.B. Romagnolo (HUEM 22,770) / Bosque Municipal de Paranavaí, Paranavaí, Paraná, Brasil. Col.: M.B. Romagnolo nº 3026. Det.: M.B. Romagnolo (HUEM 19,484). *Eugenia pyriformis* Cambess.: Estação Ecológica do Caiuá, Estrada Paranapanema, Diamante do Norte, Paraná, Brasil. Col.: G.O. Landgraf et al. nº 23. Det.: M.B. Romagnolo (HUEM 22,508) / Estação Ecológica do Caiuá, Estrada Paranapanema, Diamante do Norte, Paraná, Brasil. Col.: G.O. Landgraf et al. nº21. Det.: M.B. Romagnolo (HUEM 22,506) / Estação Ecológica do Caiuá, Diamante do Norte, Paraná, Brasil. Col.: M.B. Romagnolo nº 3276. Det.: M.B. Romagnolo (HUEM 21,736). *Eugenia langsdorffii* O.Berg: Sobradinho Distrito Federal, Brasil. Col.: G. Hatschbach nº 37187. Det.: M. Sobral (MBM 37,079) / Rodovia BR-163, Itiquira, Mato Grosso, Brazil. Col.: G. Hatschbach nº 37442. Det.: M. Sobral (MBM 64,963) / Serra do Cabral, Minas Gerais, Brasil. Col.: G. Hatschbach et al. nº 73526. Det.: M. Sobral (MBM 272,370). *Eugenia supraaxillaris* Spring.: Sete Barras, São Paulo, Brasil. Col.: L.P.C. Morellato nº 837. Det.: J. Kuntz (ESA 36,422). *Eugenia*

expansa Spring ex Mart.: Ilha do Cardoso praia de Ipanema, mata de enconsta do Morro Ipanema, Cananéia, São Paulo, Brasil. Col.: M.R.F. Melo et al. nº 564. (SORO 4,168) / Parque Estadual da Ilha do Cardoso, Cananéia, São Paulo, Brasil. Col.: J.B. Baitello nº 26. Det.: O.T. Aguiar (MBM 317,700) / Rio Serra dos Órgãos, Limoeiro, Rio de Janeiro, Brasil. Col.: P. Occhioni nº 6669. Det.: M. Sobral (MBM 32,507). *Eugenia involucrata* DC.: Estação Ecológica do Caiuá, Projeto Madeira, Diamante do Norte, Paraná, Brasil. Col.: V. Silva nº 36. Det.: M.B. Romagnolo (HUEM 26,182) / Usina da Copel, Londrina, Paraná, Brasil. Col.: Mª. Milaneze-Gutierre s/n. Det.: M. Sobral (HUEM 9,226) / Jardim Liberdade, Maringá, Paraná, Brasil. Col.: I. Maria s/n. Det.: M. Sobral (HUEM 9,225). *Eugenia acutata* Miq.: Sete Barras, São Paulo, Brasil. Col.: N.M. Ivanauskas nº 5020. Det.: F.F. Mazine (ESA 142909). *Eugenia arvensis* Vell.: Col.: K.S. Valdemarin nº 414. Det.: K.S. Valdemarin. *Eugenia subterminalis* DC.: Estação Ecológica do Caiuá, Diamante do Norte, Paraná, Brasil. Col.: Mª.C. Souza nº 3465. Det.: Mª.C. Souza (HUEM 29,139) / Parque Estadual de Guartelá, Tibagi, Paraná, Brasil. Col.: F.F.Mazine et al. nº 974. Det.: Mª.C. Souza (HUEM 11,553) / Rio Paraná, Porto Rico, Paraná, Brasil. Col.: M.B. Romagnolo nº 687. Det.: M.B Romagnolo (HUEM 9,579). *Eugenia excelsa* O.Berg.: Coruripe, Alagoas, Brasil. Col.: M.A.B.L. Machado nº 123. Det.: A. Giaretta (ESA 102,409). *Eugenia florida* DC.: Estação Ecológica de Caiuá, trilha do Mico, Diamante do Norte, Paraná, Brasil. Col.: M.B. Romagnolo nº 3245. Det.: M.B. Romagnolo (HUEM 21,652) / Ribeirão São Pedro, São Pedro do Paraná, Paraná, Brasil. Col.: M.B. Romagnolo nº 632. Det.: M.B Romagnolo (HUEM 28,340) / Parque Cinquentenário, Maringá, Paraná, Brasil. Col.: M.B. Romagnolo nº 3431. Det.: M.B. Romagnolo (HUEM 25,614). *Eugenia patens* Poir.: Les Nouragues, French Guiana. Col.: E. Lucas et al. nº 103. Det.: Det.: F.F. Mazine (ESA 142,911). *Eugenia modesta* Phil.: Parque Nacional do Caraça, Santa Bárbara, Minas Gerais, Brasil. Col.: F.F. Mazine; S. Vieira; G.O. Romão nº 852. Det.: F.F. Mazine (SORO 4,655). *Eugenia paracatuana* O.Berg: Estação Ecológica do Caiuá, estrada do trapiche, Diamante do Norte, Paraná, Brasil. Col.: M.B. Romagnolo nº 3380. Det.: M. Sobral (HUEM 25,073) / Estação Ecológica do Caiuá, Diamante do Norte, Paraná, Brasil. Col.: M.B. Romagnolo nº 3204. Det.: M. Sobral (HUEM 21,428) / Estação Ecológica do Caiuá, borda da mata, Diamante do Norte, Paraná, Brasil. Col.: M.B. Romagnolo nº 3194. Det.: M. Sobral (HUEM 21,415). *Eugenia repanda* O.Berg: Estação Ecológica do Caiuá, próximo ao Ribeirão Conceição, Diamante do Norte, Paraná, Brasil. Col.: M.B. Romagnolo nº 2021. Det.: M.B. Romagnolo (HUEM 15,955) / Bosque Municipal de Paranavaí, Paranavaí, Paraná, Brasil. Col.: M.B. Romagnolo et al. nº 3037. Det.: M.B. Romagnolo (HUEM 19,924) / Rio Paraná, São Pedro do Paraná, Paraná, Brasil. Col.: M.B. Romagnolo et al. nº 462. Det.: M.B. Romagnolo (HUEM 23,700). *Eugenia speciosa* Cambess.: Serra do Lopo, Cantos do Céu Pousada, Extrema, Minas Gerais, Brasil. Col.: nº 884. (SORO 1,225) / Serra do Lopo, Cantos do Céu Pousada, Extrema, Minas Gerais, Brasil. Col.: nº 1087 (SORO 1,226) / Picinguaba - próximo ao alojamento, Ubatuba, São Paulo, Brasil. Col.: H.F. Leitão-Filho et al., nº 34786 (SORO 4,890). *Eugenia cerasiflora* Miq.: São Miguel Arcanjo, São Paulo, Brasil. Col.: P.L.R. Moraes nº 1289. Det.: F.F. Mazine (ESA 87,192). *Eugenia bahiensis* DC.: Guarapari, Espirito Santo, Brasil. Col.: J.M.L. Gomes nº 1464. Det.: M. Sobral (VIES 5,435). *Eugenia hirta* O.Berg: Coastal Zone, Bahia, Brasil. Col.: R.M. Harley, nº 17279. Det.: M. Sobral (ESA 108,544). *Eugenia subavenia* O.Berg: Parque Estadual de Ilhabela, São Paulo, Brasil. Col.: Proença, S.L. et al. nº 84. Det.: F.F. Mazine (SORO 4,154). *Eugenia stictopetala* Mart. ex DC.: Oliveira dos Brejinhos, Bahia, Brasil. Col.: R.C. Forzza et al. nº 1236. Det.: J.E.Q Faria (SORO 6,844). *Eugenia goiapabana* Sobral & Mazine: Santa Teresa, Espírito Santo, Brasil. Col.:

J.E.Q. Faria; V.G. Staggemeier nº 2481. Det.: J.E.Q. Faria (HUFSJ 5,792). *Eugenia astringens* Cambess.: Ilha do Cardoso, Cananéia, São Paulo, Brasil. Col.: F. de Barros nº 722. Det.: O.T. Aguiar (SORO 3,383, HUFSJ 6,559). *Eugenia pluriflora* DC.: Sorocaba, São Paulo, Brasil. Col.: J. F. Almeida et al. nº 18. Det.: F.F. Mazine (SORO 4,832). *Eugenia leptoclada* O.Berg: Cunha, São Paulo, Brasil. Col.: E. J. Lucas et al. nº 389. Det.: F.F. Mazine (ESA 53,389). *Eugenia neoverrucosa* Sobral: Estação Ecológica do Caiuá, Trilha do Mico, Diamante do Norte, Paraná, Brasil. Col.: G.O. Landgraf et al. nº 31. Det.: M.B. Romagnolo (HUEM 22,516) / Estação Ecológica do Caiuá, Trilha do Mico, Diamante do Norte, Paraná, Brasil. Col.: M.B. Romagnolo nº 3242. Det.: M.B. Romagnolo (HUEM 21,648) / Estação Ecológica do Caiuá, Trilha da Cachoeira, Diamante do Norte, Paraná, Brasil. Col.: M.B. Romagnolo nº 3367. Det.: M.B. Romagnolo (HUEM 23,760). *Eugenia egensis* DC.: Rio Paraná, canal cortado, margem esquerda, Porto Rico, Paraná, Brasil. Col.: L.M. Garcia et al. nº 770. Det.: M.B. Romagnolo (HUEM 25,207) / Rio Paraná, Mato Grosso do Sul, Brasil. Col.: M.B. Romagnolo nº 388. Det.: M.B. Romagnolo (HUEM 21,471) / Bosque Municipal de Paranavaí, Paranavaí, Paraná, Brasil. Col.: M.B. Romagnolo nº 3041. Det.: M.B. Romagnolo (HUEM 19,891). *Eugenia flavescens* DC.: Caracol, Piauí, Brasil. Col.: G. Martinelli; E. Fernandes nº 16270. Det.: M. Sobral (SORO 4,358). *Eugenia batingabranca* Sobral: Linhares, Espírito Santo, Brasil. Col.: DA Folli nº 7073. Det.: A. F. Domingos (CVRD 14,450). *Eugenia dodonaeifolia* Cambess.: São Paulo, São Paulo, Brasil. Col.: F.C. Hoehne nº 28417. Det.: M.L. Kawasaki (ESA 72,854). *Eugenia gracillima* Kiaersk: Estação Ecológica de Caiuá, Trilha do Mico, Diamante do Norte, Paraná, Brasil. Col.: M.B. Romagnolo nº 3246. Det.: M.B. Romagnolo (HUEM 21,653) / Estação Ecológica de Caiuá, borda da mata, Diamante do Norte, Paraná, Brasil. Col.: M.B. Romagnolo nº 3197. Det.: M.B. Romagnolo (HUEM 21,417) / Estação Ecológica de Caiuá, Estrada Ribeirão Conceição, Diamante do Norte, Paraná, Brasil. Col.: M.B. Romagnolo nº 3164. Det.: M.B. Romagnolo (HUEM 19,556). *Eugenia hiemalis* Cambess.: Rio Areia, margem direita, Marilena, Paraná, Brasil. Col.: C.E. Bento Fernandes nº 36. Det.: M.B. Romagnolo (HUEM 21,180) / Estação Ecológica do Caiuá, Estrada Paranapanema, Diamante do Norte, Paraná, Brasil. Col.: G.O. Landgraf nº 14. Det: M.B. Romagnolo (HUEM 22,499) / Rio Samambaia, margem direita, próximo a ponte, Batayporã, Mato Grosso do Sul, Brasil. Col.: M.M. Pulzatto nº 03. Det.: M.B. Romagnolo (HUEM 23,157). *Eugenia mosenii* (Kausel) Sobral: Cananéia, São Paulo, Brasil. Col.: Barros L. et al. nº 1479. Det.: O.T. Aguiar (SORO 1,072). *Eugenia ramboi* D. Legrand.: Estação Ecológica do Caiuá, próximo a ponte do Ribeirão Diamante, Diamante do Norte, Paraná, Brasil. Col.: M.B. Romagnolo nº 3417 (HUEM 25,404) / Estação Ecológica do Caiuá, estrada próxima ao Ribeirão Diamante, Diamante do Norte, Paraná, Brasil. Col.: M.B. Romagnolo nº 3265. Det.: M.B. Romagnolo (HUEM 21,713) / Ribeirão São Pedro, São Pedro do Paraná, Paraná, Brasil. Col.: M.B. Romagnolo nº 23. Det.: M.B. Romagnolo (HUEM 26,832).
