# Supplementary material for: The anatomy of the seed-coat includes diagnostic characters in the subtribe Eugeniinae (Myrteae, Myrtaceae)
Source: Front Plant Sci. 2022 Oct 5;13:981884. doi: 10.3389/fpls.2022.981884 (PMC9580042; doi:10.3389/fpls.2022.981884)
Supplement: Supplementary file 2 [file Table_1.pdf]

Supplementary Table 1. Potentially informative anatomical characters of the ovule and the mature seed of species of the Subtribe Eugeniinae (original data) and Myrteae subtribes *sensu* Lucas et al. (2019) [taken from Narayanaswasm and Roy, 1960[+]; Corner, 1976[++]; Van Wyk and Botha, 1984[+++]; Ciccarelli et al., 2005[++++]; Moreira-Coneglian, 2007*; Moreira-Coneglian, 2011**; Machado, 2014***; Galan, 2020****]. Notes: Some characters described by these authors were changed and other characteristics were included from the observation of the illustrations of these works in order to standardize the description and facilitate comparison. Key: ?, unknown; -, inapplicable.

**Subtribe**	**Section**	**Species**	**Ovule type/ Integument number**	**Integument layers**		**Mature seed**				**Seed type**
				**Outer**	**Inner**	**Testa**			**Tegmen**	
						**Exotesta**	**Mesotesta**	**Endotesta**		
		Myrcianthes pungens (O.Berg) D.Legrand	Anatropous → Campylotropous/ Bitegmic	3	2	Tabular-cuboid obliquely elongated, thick-walled non-lignified cells, palisade of lignified macrosclereids in the raphe	Crushed	Crushed	Crushed	Partially exotestal/ Perichalazal
		Eugenia arenosa Mattos	Campylotropous/ Unitegmic	6	–	Tabular-cuboid obliquely elongated, thin-walled cells	Outer: aerenchymatous/ Inner: strongly lignified fiber-like cells	Strongly lignified fiber-like cells	–	Inner mesotestal/ Pachychalazal
**Eugeniinae**	*Eugenia* sect. *Pseudeugenia* Faria	*E. klotzchiana* O.Berg	?	?	?	Tabular-cuboid obliquely elongated, thin-walled cells	Outer: aerenchymatous/ Inner: lignified fiber-like cells	Lignified cuboid cells	?	Inner mesotestal/ ?
		E. dysenterica DC.	Campylotropous/ Unitegmic	6	–	?	?	?	-	?
	E. sect. *Hexachlamys* (O.Berg) Mattos	*E. myrcianthes* Nied.	Campylotropous/ Bitegmic	3-5	2-3	Tabular-cuboid, thin-walled cells	Outer: aerenchymatous/ Inner: strongly lignified fiber-like cells	Crushed	Crushed	Inner mesotestal/ Pachychalazal
		E. brasiliensis Lam.	Campylotropous/ Bitegmic	6	2	Tabular-cuboid obliquely elongated , thin-walled cells	Aerenchymatous	Crushed	Crushed	Non-lignified/ Pachychalazal
		E. longipedunculata	Campylotropous/	4	2	Tabular obliquely	Aerenchymatous	Crushed	Crushed	Non-lignified/

E. sect. *Eugenia*	Nied.	Bitegmic			elongated, thin-walled cells				Pachychalazal
	E. pitanga Kiaersk.***	Campylotropous/ Bitegmic	4	2	Tabular-cuboid obliquely elongated, thin-walled cells	Outer: aerenchymatous/ Inner: non-lignified fiber-like cells	Crushed	Crushed	Non-lignified/ Pachychalaza
	E. uniflora L.	Campylotropous/ Unitegmic	3-5	-	Tabular-cuboid obliquely elongated, thin-walled cells	Aerenchymatous	Crushed	-	Non-lignified/ Pachychalaza
E. sect. *Pilothecium (*Kiaersk.) D.Legrand	*E. pyriformis* Cambess.	Campylotropous/ Bitegmic	3-5	2	Tabular-cuboid obliquely elongated, thin-walled cells, palisade of lignified macrosclereids in the micropyle	Aerenchymatous	Crushed	Crushed	Non-lignified/ Pachychalaza
	E. langsdorffii O.Berg	Campylotropous/ Bitegmic	3-5	2-3	Tabular-cuboid thin-walled cells	Parenchymatic: groups of sclereids	Crushed	Crushed	Mesotestal
	E. supraaxillaris Spring.	-	?	?	Tabular-cuboid thin-walled cells	Parenchymatic: groups of sclereids	Crushed	?	Mesotestal
E. sect. *Phyllocalyx* Nied.	*E. expansa* Spring ex Mart.	Campylotropous/ Bitegmic	3-5	2-3	?	?	?	?	?
	E. involucrata DC.	Campylotropous/ Bitegmic	3-5	2	Tabular-cuboid obliquely elongated, thin-walled cells	Aerenchymatous/ Last layers of with thin-walled cells	Thin-walled cells	Crushed	Non-lignified/ Pachychalaza
E. sect. *Schizocalomyrtus* (Kausel) Mattos	*E. acutata* Miq.	?	?	?	Tabular obliquely elongated, thin-walled cells	Outer: aerenchymatous (groups of sclereids); Inner: parenchymatic	Thin-walled cells	Crushed	Outer mesotestal/ ?
	E. arvensis Vell.	?	?	?	Tabular obliquely elongated, thin-walled cells	Parenchymatic/ Inner: groups of sclereids	Thick-walled cells	?	Inner meso-endotestal/ ?
	E. subterminalis DC.	Campylotropous/ Unitegmic	3-5	-	Palisade of radially elongated lignified macroesclereids or cuboid sclereids	Outer: aerenchymatous/ Inner: non-lignified fiber-like cells	Crushed	-	Exotestal/ Pachychalaza
E. sect. *Excelsae* Mazine & E.Lucas	*E. excelsa* O.Berg	?	?	?	Palisade of radially elongated lignified macroesclereids	Outer: aerenchymatous/ Inner: groups of sclereids	Crushed	?	Exomesotestal/ ?

	E. simii Dümmer+++	Campylotropous/ Bitegmic	4-6	2	Palisade of radially elongated lignified macroesclereids	Lignified fiber-like cells	Crushed	Crushed	Exomesotestal
E. sect. *Jossinia* (DC.) Nied. Group X	*E. capensis* Harv.+++	Campylotropous/ Bitegmic	4-6	2	Palisade of radially elongated non-lignified cells	Non-lignified fiber-like cells	Crushed	Crushed	Non-lignified
	E. natalitia Sond.+++	Campylotropous/ Bitegmic	4-6	2	Palisade of radially elongated lignified macroesclereids	Non-lignified fiber-like cells	Crushed	Crushed	Exotestal
	E. umtamvunensis A.E.van Wyk+++	Campylotropous/ Bitegmic	4-6	2	Palisade of radially elongated lignified macroesclereids	Lignified fiber-like cells	Crushed	Crushed	Exomesotestal
	E. zeyheri Harv.+++	Campylotropous/ Bitegmic	4-6	2	Palisade of radially elongated lignified macroesclereids	Lignified fiber-like cells	Crushed	Crushed	Exomesotestal/ Partly pachychalazal
	E. albanensis Sond.+++	Campylotropous/ Bitegmic	4-6	2	Lignified fiber-like cells	Lignified fiber-like cells	Crushed	Crushed	Exomesotestal/ Partly pachychalazal
	E. erythrophylla Strey+++	Campylotropous/ Bitegmic	4-6	2	Lignified fiber-like cells	Lignified fiber-like cells	Crushed	Crushed	Exomesotestal/ Partly pachychalazal
	E. verdoornieae A.E.van Wyk+++	Campylotropous/ Bitegmic	4-6	2	Lignified fiber-like cells	Lignified fiber-like cells	Crushed	Crushed	Exomesotestal/ Partly pachychalazal
E. sect. *Jossinia* (DC.) Nied. Group Y	*E. woodii* Dümmer+++	Campylotropous/ Bitegmic	4-6	2	Lignified fiber-like cells	Lignified fiber-like cells	Crushed	Crushed	Exomesotestal/ Partly pachychalazal
	E. zuluensis Dümmer+++	Campylotropous/ Bitegmic	4-6	2	Palisade of radially elongated lignified macroesclereids	Lignified fiber-like cells	Crushed	Crushed	Exomesotestal/ Partly pachychalazal
	E. sp A.+++	Campylotropous/ Bitegmic	4-6	2	Lignified fiber-like cells	Lignified fiber-like cells	Crushed	Crushed	Exomesotestal/ Partly pachychalazal
	E. sp. B+++	Campylotropous/ Bitegmic	4-6	2	Lignified fiber-like cells	Lignified fiber-like cells	Crushed	Crushed	Exomesotestal/ Partly pachychalazal
	E. sp. C+++	Campylotropous/ Bitegmic	4-6	2	Lignified fiber-like cells	Lignified fiber-like cells	Crushed	Crushed	Exomesotestal/ Partly pachychalazal
	E. florida DC.	Campylotropous/ Unitegmic	6	-	Palisade of radially elongated lignified macroesclereids	Parenchymatic/ Inner: groups of sclereids	Crushed	-	Exomesotestal/ Pachychalaza
E. sect. *Racemosae* O.Berg	*E. patens* Poir.	?	?	?	Tabular obliquely elongated, thin-walled cells	Crushed	Crushed	?	Non-lignified
	E. modesta DC.	?	?	?	Palisade of radially	Outer:	Crushed	?	Exotestal

					elongated lignified macroesclereids or cuboid sclereids	aerenchymatous (crushed)/ Inner: parenchymatic (groups of sclereids)			
	E. paracatuana O.Berg	Campylotropous/ Bitegmic	3-4	2	Palisade of radially elongated lignified macroesclereids	Outer: crushed/ Inner: non-lignified fiber-like cells	Crushed	Crushed	Exotestal/ Pachychalaza
	E. repanda O.Berg	Campylotropous/ Bitegmic	3-4	2	Tabular-cuboid obliquely elongated, thin-walled cells	Crushed	Crushed	Crushed	Non-lignified/ Pachychalaza
E. sect. *Speciosae* Bünger & Mazine	*E. speciosa* Cambess.	Campylotropous/ Bitegmic	3-5	2	Tabular-cuboid obliquely elongated, thin-walled cells	Outer: crushed/ Inner: lignified fiber-like cells	Lignified fiber-like cells	Crushed	Inner mesotestal/ Pachychalaza
	E. punicifolia (Kunth) DC.*	Campylotropous/ Bitegmic	3-4	2-3	Palisade of radially elongated lignified macroesclereids or cuboid sclereids	Lignified fiber-like cells	Lignified fiber-like cells	Crushed	Testal/ Pachychalaza
	E. bahiensis DC.	?	?	?	Palisade of radially elongated lignified macroesclereids	Lignified fiber-like or isodiametric cells	Lignified fiber-like or isodiametric cells	?	Testal/ ?
	E. hirta O.Berg	?	?	?	Palisade of radially elongated lignified macroesclereids	Lignified fiber-like or isodiametric cells	Lignified fiber-like or isodiametric cells	?	Testal/ ?
E. sect. *Umbellatae* O.Berg	*E. subavenia* O.Berg	?	?	?	Palisade of radially elongated lignified macroesclereids	Outer: parenchymatic/ Inner: lignified fiber-like or isodiametric cells	Lignified isodiametric cells	?	Exomesotestal/ ?
	E. stictopetala Mart. ex DC.				Palisade of radially elongated lignified macroesclereids	Lignified isodiametric cells	Lignified isodiametric cells		Testal/ ?
	E. bimarginata DC.**	Campylotropous/ Bitegmic	3-4	2	Palisade of radially elongated lignified macroesclereids	Lignified fiber-like cells	Lignified fiber-like cells	Crushed	Testal/ Pachychalaza
	E. pluriflora DC.	?	?	?	Palisade of radially elongated lignified macroesclereids	Outer: lignified isodiametric cells/ Inner: thin-walled isodiametric cells	Thin-walled isodiametric cells	?	Exomesotestal/ ?
	E. leptoclada O.Berg	?	?	?	Palisade of radially elongated lignified macroesclereids	Non-lignified isodiametric and fiber-like mesotestal	Thin-walled, tabular cells	?	Exotestal/ ?

						cells			
	E. neoverrucosa Sobral	Campylotropous/ Bitegmic	3-4	2	Palisade of radially elongated lignified macroesclereids	Parenchymatic	Thin-walled cells	Crushed	Exotestal/ Pachychalaza
	E. egensis DC.	Campylotropous/ Unitegmic	6	-	Palisade of radially elongated lignified macroesclereids	Outer: lignified fiber-like cells/ Inner: thin-walled cells	Thin-walled cells	-	Exotestal/ Pachychalaza
	E. flavescens DC.	?	?	?	Palisade of radially elongated lignified macroesclereids	Lignified isodiametric cells	Lignified isodiametric cells	Crushed	Testal/ ?
	E. aurata O.Berg**	Campylotropous/ Bitegmic	3-4	2	Palisade of radially elongated lignified macroesclereids	Lignified fiber-like cells	Lignified fiber-like cells	Crushed	Testal// Pachychalaza
	E. batingabranca Sobral	?	?	?	Palisade of radially elongated lignified macroesclereids	Non-lignified fiber-like cells	Non-lignified fiber-like cells	?	Exotestal/ ?
	E. gracillima Kiaersk.	Campylotropous/ Bitegmic	2-3	2	Tabular, thin-walled cells	Outer: crushed/ Inner: lignified fiber-like cells	Crushed	Crushed	Inner mesotestal/ Perichalaza
	E. hiemalis Cambess.	Campylotropous/ Bitegmic	3-4	2	Palisade of radially elongated lignified macroesclereids or cuboid sclereids	Lignified fiber-like cells	Thick-walled cuboid-tabular cells	Crushed	Testal/ Pachychalaza
	E. mosenii (Kausel) Sobral	?	?	?	Lignified cuboid sclereids	Outer:crushed/ Inner: lignified isodiametric and fiber-like cells	Thick-walled cuboid-tabular cells	?	Testal/ ?
	E. ramboi D.Legrand	Campylotropous/ Bitegmic	3-4	2	Obliquely elongated lignified cuboid sclereids	Lignified fiber-like cells	Lignified fiber-like cells	Crushed	Testal/ Pachychalaza
**Pimentinae**	*Psidium guajava* L.[+]	Anatropous/ Bitegmic	2-5	2	Cuboid, thin-walled cells	Outer: a few cell layers are thin-walled and flattened out/ Inner: the cells of the remaining layers undergo lignification	Lignified	Crushed	Inner mesotestal
	P. cujavillus Burm.f.[+]	Hemi-anatropous/ Bitegmic	2-5	2	Cuboid, thin-walled cells	Outer: a few cells layers are thin-walled and flattened out/ Inner: the cells of the remaining layers	Lignified	Crushed	Inner mesotestal

						undergo lignification				
	P. cattleyanum Sabine[++]	Anatropous/ Bitegmic	2-4	2	Non-palisade, thin-walled cells	Outer: thin-walled cells (2-6 cells layers), eventually more or less crushed/ Inner: Sclerotic thick-walled, closely pitted, lignified (wide lumen)	Crushed	Crushed	Inner mesotestal	
	P. australe var. *argenteum* (O.Berg) Landrum[**]	Anatropous/ Bitegmic	3	2	Tabular-cuboid, thin-walled cells	Outer: thin-walled radially elongated cells (sarcotesta)/Inner: thick-walled, lignified cells (sclereids)	Lignified	Crushed	Inner mesotestal	
	P. guianense Pers.[**]	Anatropous/ Bitegmic	3	2	Tabular-cuboid, thin-walled cells	Outer: thin-walled radially elongated cells cells (sarcotesta)/Inner: thick-walled, lignified cells (sclereids)	Lignified (sclereids)	Crushed	Inner Mesotestal	
	Campomanesia adamantium (Cambess.) O.Berg***	Campylotropous/ Bitegmic	3	2	Radially elongated, thin-walled cells	Crushed	Crushed	Crushed	Non-lignified/ Pachychalaza	
	Campomanesia pubescens O.Berg*	Campylotropous/ Bitegmic	3-4	2	Cuboid lignified sclereids	Some sclereids in mesotesta/Rest of mesotesta crushed	Crushed	Crushed	Exotestal/ Perichalaza	
**Ugninae**	?	?	?	?	?	?	?	?	?	
**Luminae**	*Myrceugenia alpigena* (DC.) Landrum***	Campylotropous/ Bitegmic	2	2	Tabular, thin-walled cells	Crushed	Crushed	Crushed	Non-lignified	
	Myrcia bella Cambess.**	Campylotropous/ Bitegmic	3	2	Palisade of radially elongated lignified macroesclereids	Crushed	Crushed	Crushed	Exotestal/ Perichalaza	
**Myrciinae**	*M. laruotteana* Cambess.**	Campylotropous/ Bitegmic	3	2	Palisade of radially elongated lignified macroesclereids	Crushed	Crushed	Crushed	Exotestal/ Perichalaza	
	M. multiflora DC.***	Campylotropous/ Bitegmic	2	2	Palisade of radially elongated lignified	Crushed	Crushed	Crushed	Exotestal/ Pachychalaza	

					macroesclereids				
	Siphoneugena reitzii D.Legrand****	Campylotropous/ Bitegmic	2	2	Cuboid lignified cells	Outer: isodiametric lignified cells/ Inner: crushed	Crushed	Crushed	Exomesotestal/ Perichalaza
	S. widgreniana O.Berg****	Campylotropous/ Bitegmic	2	2	Cuboid lignified cells	Outer: isodiametric lignified cells/ Inner: crushed	Crushed	Chushed	Exomesotestal/ Perichalaza
	Neomitranthes gemballae (D.Legrand) D.Legrand****	Campylotropous/ Bitegmic	3-4	2	Cuboid, thin-walled cells	Outer: isodiametric, thin-walled cells/ Iinner: crushed	Crushed	Crushed	Non-lignified/ Perichalaza
	N. glomerata (D.Legrand) D.Legrand****	Campylotropous/ Bitegmic	3-4	2	Cuboid, thin-walled cells	Outer: isodismetric, thin-walled cells/ Iinner: crushed	Crushed	Crushed	Non-lignified/ Perichalaza
	Plinia brachybotrya (D.Legrand) Sobral****	Campylotropous/ Bitegmic	2	2	Cuboid, thin-walled cells	Outer: isodiametric, thin-walled cells/ Iinner: crushed	Crushed	Crushed	Non-lignified/ Perichalaza
**Pliniinae**	*P. cauliflora* (Mart.) Kausel****	Campylotropous/ Bitegmic	2	2	Cuboid, thin-walled cells	Outer: isodiametric, thin-walled cells Iinner: crushed	Crushed	Crushed	Non-lignified/ Perichalaza
	Myrciaria delicatula (DC.) O.Berg**	Campylotropous/ Bitegmic	2	2	Cuboid, thick-walled cells	Outer: isodiametric, thick-walled cells/ Inner: crushed	Crushed	Crushed	Exomesotestal/ Pachychalaza
	M. floribunda O.Berg****	Campylotropous/ Bitegmic	2	2	Palisade of radially elongated lignified macroesclereids	Crushed	Crushed	Crushed	Exotestal/ Perichalaza
	M. glomerata O.Berg****	Campylotropous/ Bitegmic	3	2	Cubloid, non-lignified cells	Crushed	Crushed	Crushed	Non-lignified/ Perichalaza
	Algrizea macrochlamys (DC.) Proença & NicLugh.****	Campylotropous/ Bitegmic	3-4	2	Palisade of radially elongated lignified macroesclereids	Crushed	Crushed	Crushed	Exotestal/ Perichalaza
	A. minor Sobral, Faria & Proença****	Campylotropous/ Bitegmic	3-4	2	Palisade of radially elongated lignified macroesclereids	Crushed	Crushed	Crushed	Exotestal/ Perichalaza
**Blepharocalycinae**	*Blepharocalyx*	Anfitropous/	2	2	Tabular-cuboid,	Crushed	Crushed	Crushed	Non-lignified/

	salicifolius (Kunth) O.Berg**	Bitegmic			thin-walled wider cells				Perichalaza
**Myrtinae**	*Myrtus communis* L.[++++]	Anatropous/ Bitegmic	2-3	2	Tabular-cuboid lignified cells	Isodiametrics lignified cells	Cuboid lignified cells	Outer: lignified cells/ Inner: thin-walled cells	Testal, exomesotegmic
	Rhodomyrtus tomentosa Wight[++]	Campylotropous/ Bitegmic	3-4	2	Largest cells (bulging outer wall)	Outer: lignified thick-walled enlargest cells/ Inner: row of cells least enlarged	Thin-walled cuboid cells	Crushed	Exomesotestal
**Decaspeminae**	*Decaspermum fruticosum* J.R.Forst & G.Forst[++]	Campylotropous/ Bitegmic	2-3	2	Lignified cells, strongly radially elongated in groups	Small radially compressed cells with thin but slightly lignified walls	Small radially compressed cells with thin but slightly lignified walls	Crushed	Testal
	Rhodamnia cinerea Jack[++]	Campylotropous/ Bitegmic	2-3	2	Lignified cells, no-radially elongated	Small radially compressed cells with thin but slightly lignified walls	Small radially compressed cells with thin but slightly lignified walls	Longitudinally oblong endotegmic cells, firm non- lignified walls (yellowish brown)	Testal
