# Supplementary material for: The anatomy of the seed-coat includes diagnostic characters in the subtribe Eugeniinae (Myrteae, Myrtaceae)
Source: Front Plant Sci. 2022 Oct 5;13:981884. doi: 10.3389/fpls.2022.981884 (PMC9580042; doi:10.3389/fpls.2022.981884)
Supplement: Supplementary file 3 [file Table_2.pdf]

Supplementary Table 2. Scores for potentially 13 informative anatomical character states of the ovule and the mature seed of Subtribe Eugeniinae and Myrteae species (original data and taken from Corner, 1976[+]; Van Wyk and Botha, 1984[++]; Moreira-Coneglian, 2007*; Moreira-Coneglian, 2011**; Machado, 2014***; Galan, 2020****). Key: ?, unknown; -, inapplicable.

Subtribe	Section	Species	1	2	3	4	5	6	7	8	9	10	11	12
**Eugeniinae**		*Myrcianthes pungens* (O.Berg) D.Legrand	0	1	0	0	1	1	1	1	1	1	1	1
	Eugenia sect. *Pseudeugenia* Faria	*Eugenia arenosa* Mattos	1	0	1	-	1	0	-	0	0	0	2	0
		E. klotzchiana O. Berg	?	?	?	?	1	0	-	0	0	0	2	?
		E. dysenterica DC.	1	0	1	-	?	?	?	?	?	?	?	?
	E. sect. *Hexachlamys* (O.Berg) Mattos	*E. myrcianthes* Nied.	1	1	1	1	0	0	-	0	0	1	2	0
	E. sect. *Eugenia*	*E. brasiliensis* Lam.	1	1	1	0	1	0	-	0	1	1	0	0
		E. longipedunculata Nied.	1	1	1	0	1	0	-	0	1	1	0	0
		E. pitanga Kiaersk.***	1	1	1	0	1	0	-	0	1	1	0	0
		E. uniflora L.	1	0	1	-	1	0	-	0	1	1	0	0
	E. sect. *Pilothecium (*Kiaersk.) D.Legrand	*E. pyriformis* Cambess.	1	1	1	0	1	0	2	0	1	1	0	0
		E. langsdorffii O.Berg	1	1	1	1	0	0	-	1	0	1	2	?
		E. supraaxillaris Spring.	?	?	?	?	0	0	-	1	0	1	2	?
	E. sect. *Phyllocalyx* Nied.	*E. expansa* Spring ex Mart.	1	1	1	1	?	?	?	?	?	?	?	?
		E. involucrata DC.	1	1	1	0	1	0	-	0	1	1	0	0
	E. sect. *Schizocalomyrtus* (Kausel) Mattos	*E. acutata* Miq.	?	?	?	?	1	0	-	0	0	1	2	?
		E. arvensis Vell.	?	?	?	?	1	0	-	1	0	1	2	?
		E. subterminalis DC.	1	0	1	-	2	2	0	0	1	1	1	0
	E. sect. *Excelsae* Mazine & E.Lucas	*E. excelsa* O. Berg	?	?	?	?	2	2	0	0	0	1	3	?
	E. sect. *Jossinia* (DC.) Nied. Group X	*E. simii* Dümmer[++]	1	1	1	0	2	2	0	1	0	1	3	1
		E. capensis Harv.[++]	1	1	1	0	2	1	-	1	1	1	0	1
		E. natalitia Sond.[++]	1	1	1	0	2	2	0	1	1	1	1	1
		E. umtamvunensis A.E.van Wyk[++]	1	1	1	0	2	2	0	1	0	1	3	1
	E. sect. *Jossinia* (DC.) Nied. Group Y	*E. zeyheri* Harv.[++]	1	1	1	0	2	2	0	1	0	1	3	0
		E. albanensis Sond.[++]	1	1	1	0	1	2	0	1	0	1	3	0
		E. erythrophylla Strey[++]	1	1	1	0	1	2	0	1	0	1	3	0
		E. verdoornieae A.E.van Wyk[++]	1	1	1	0	1	2	0	1	0	1	3	0
		E. woodii Dümmer[++]	1	1	1	0	1	2	0	1	0	1	3	0
		E. zuluensis Dümmer[++]	1	1	1	0	2	2	0	1	0	1	3	0
		E. sp A.[++]	1	1	1	0	1	2	0	1	0	1	3	0
		E. sp. B[++]	1	1	1	0	1	2	0	1	0	1	3	0
		E. sp. C[++]	1	1	1	0	1	2	0	1	0	1	3	0
	E. sect. *Racemosae* O.Berg	*E. florida* DC.	1	0	1	-	2	2	0	1	0	1	3	0
		E. patens Poir.	?	?	?	?	1	0	-	1	1	1	0	?
		E. modesta DC.	?	?	?	?	2	2	0	0	0	1	1	?
		E. paracatuana O.Berg	1	1	1	0	2	2	0	1	1	1	1	0
		E. repanda O.Berg	1	1	1	0	0	0	-	1	1	1	0	0

	E. sect. *Speciosae* Bünger & Mazine	*E. speciosa* Cambess.	1	1	1	0	0	0	-	1	0	0	2	0
		E. punicifolia (Kunth) DC.*	1	1	1	1	2	2	0	1	0	0	3	0
		E. bahiensis DC.	?	?	?	?	2	2	0	1	0	0	3	?
		E. hirta O.Berg	?	?	?	?	2	2	0	1	0	0	3	?
		E. subavenia O.Berg	?	?	?	?	2	2	0	1	0	0	3	?
		E. stictopetala Mart. ex DC.	?	?	?	?	2	2	0	1	0	0	3	?
		E. bimarginata DC.**	1	1	1	0	2	2	0	1	0	0	3	0
		E. pluriflora DC.	?	?	?	?	2	2	0	1	0	1	3	?
	E. sect. *Umbellatae* O.Berg	*E. leptoclada* O.Berg	?	?	?	?	2	2	0	1	1	1	1	?
		E. neoverrucosa Sobral	1	1	1	0	2	2	0	1	1	1	1	0
		E. egensis DC.	1	0	1	-	2	2	0	1	0	1	1	0
		E. flavescens DC.	?	?	?	?	2	2	0	1	0	0	3	?
		E. aurata O.Berg**	1	1	1	0	2	2	0	1	0	0	3	0
		E. batingabranca Sobral	?	?	?	?	2	2	0	1	1	1	1	?
		E. gracillima Kiaersk.	1	1	0	0	0	0	-	1	0	1	2	1
		E. hiemalis Cambess.	1	1	1	0	2	2	0	1	0	1	3	0
		E. mosenii (Kausel) Sobral	?	?	?	?	0	2	0	1	0	1	3	?
		E. ramboi D.Legrand	1	1	1	0	1	2	0	1	0	0	3	0
		P. cattleyanum Sabine[+]	0	1	1	0	0	0	-	1	0	1	2	-
**Pimentinae**		*Campomanesia adamantium* (Cambess.) O.Berg***	1	1	0	0	2	0	-	1	1	1	0	0
**Luminae**		*Myrceugenia alpigena* (DC.) Landrum***	1	1	0	0	0	0	-	1	1	1	0	-
**Myrciinae**		*M. laruotteana* Cambess.**	1	1	0	1	2	2	0	1	1	1	1	1
**Pliniinae**		*Algrizea macrochlamys* (DC.) Proença & NicLugh.****	1	1	1	0	2	2	0	1	1	1	1	1

Character states used in anatomical analysis of the ovule and the mature seed-coat of Subtribe Eugeniinae *sensu* Mazine et al. (2018).

Ovule

1. Curvature : Anatropous → Campylotropous (0), Campylotropous (1)

2. Integuments number: Unitegmic (0), Bitegmic (1)

3. Outer integument layers: few (≤ 3) (0), many ≥ 3 (1)

4. Inner integument layers: two (0), two-three (1)

Mature seed

5. Exotesta cell shape: tabular and/or cuboid (0), tabular and/or cuboid obliquely elongated (fiber-like) (1), radially elongated (2)

6. Exotesta cell wall: thin (0), thick non-lignified (1), thick lignified (2)

7. Exotesta cells (macroesclereids, cuboid sclereids, lignified fiber-like cells): all seed (0), in the rapheal region (1), in the micropyle (2)

8. Aerenchymatous mesotesta: present (0), absent (1)

9. Outer and/or inner lignified mesotesta: present (0), absent (1)

10. Lignified endotesta: present (0), absent (1)

11. Mature seed-coat: non-lignified (0), exotestal (1), mesotestal (2), exomesotestal or testal (3)

12. Chalaza: pachychlaza (0), perichalaza (1)
