# Supplementary figures and images for: The anatomy of the seed-coat includes diagnostic characters in the subtribe Eugeniinae (Myrteae, Myrtaceae)
Source: Front Plant Sci. 2022 Oct 5;13:981884. doi: 10.3389/fpls.2022.981884 (PMC9580042; doi:10.3389/fpls.2022.981884)

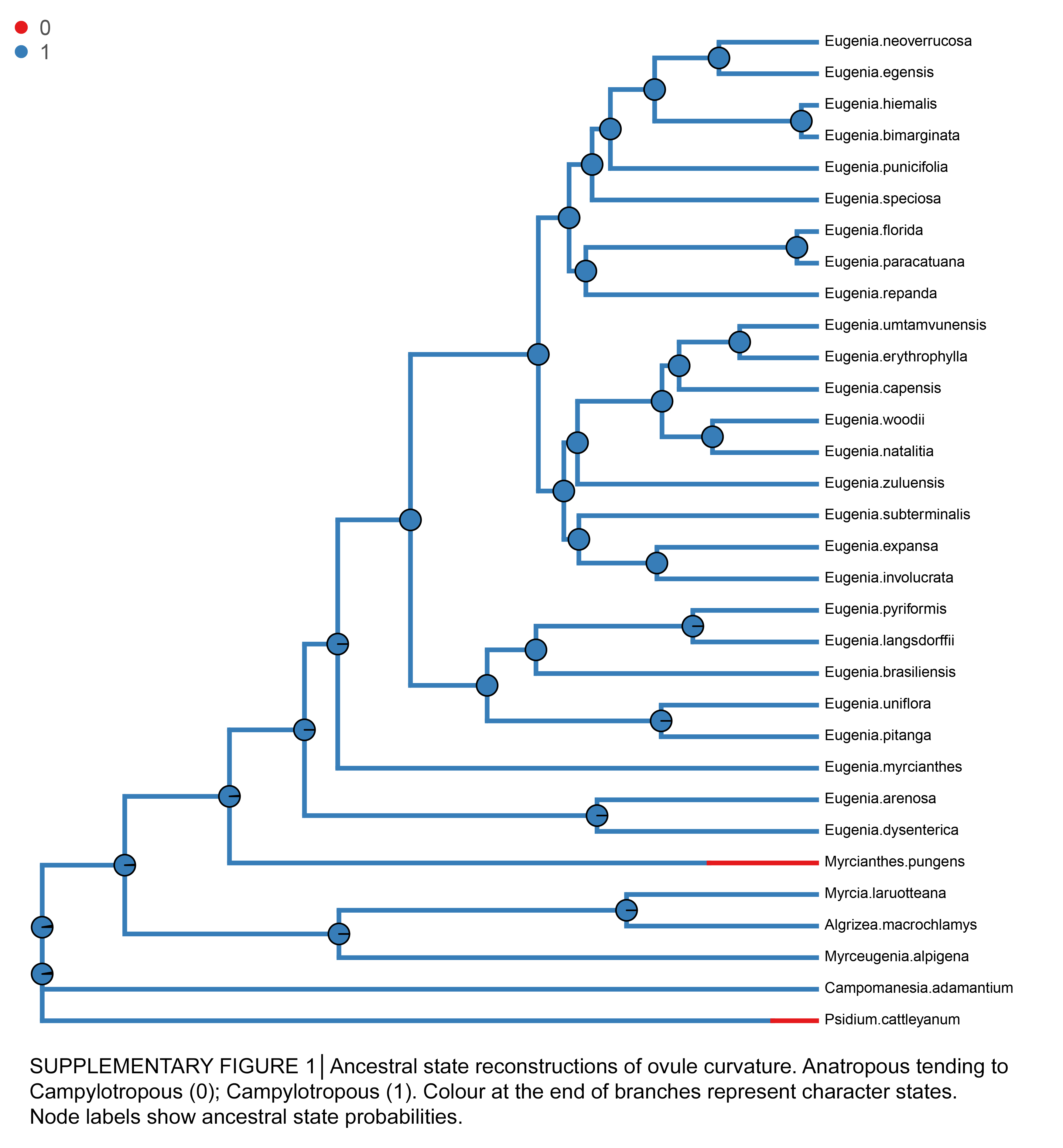

Supplement: Supplementary file 4 [file Image_1.jpg]

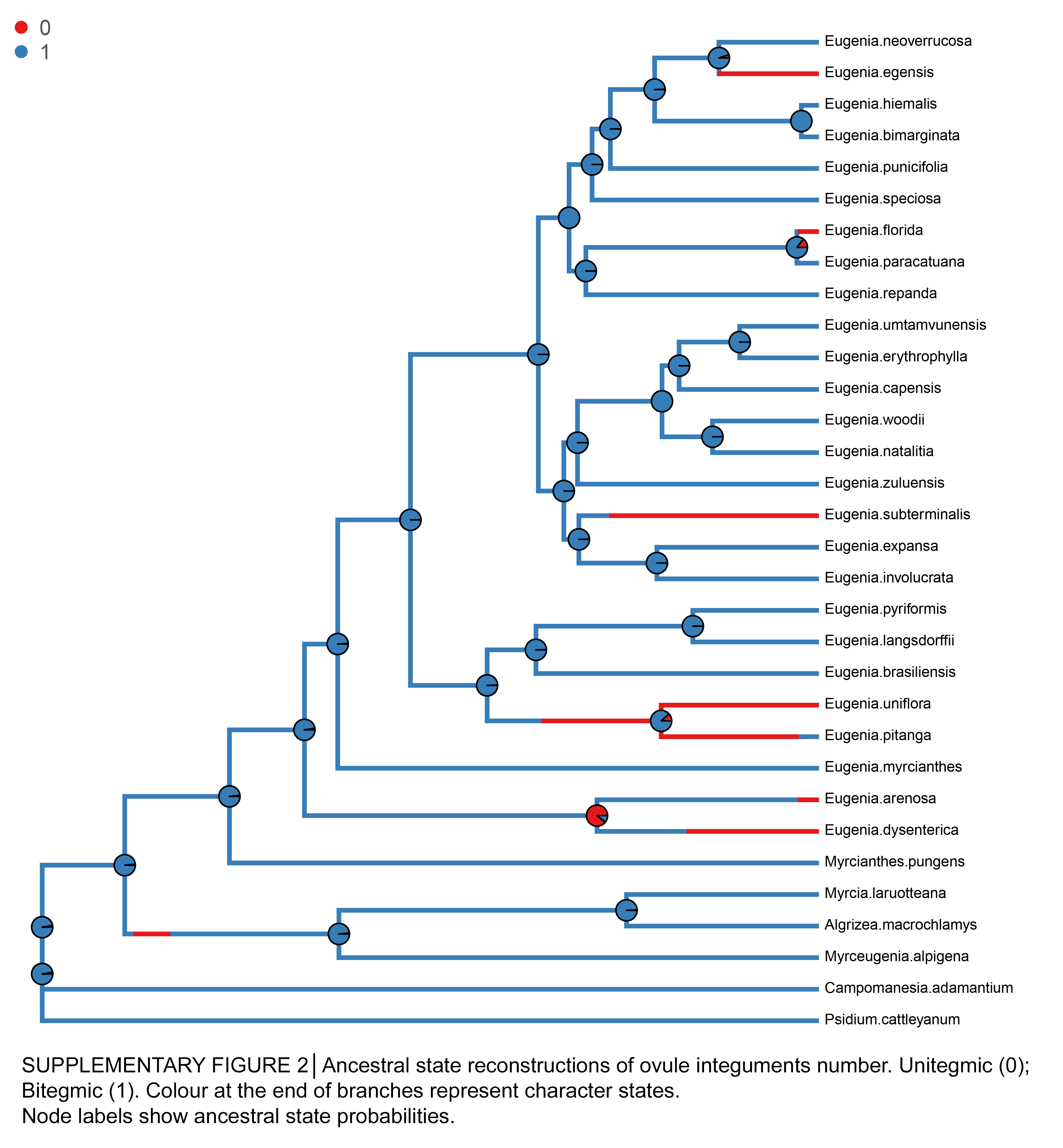

Supplement: Supplementary file 5 [file Image_2.jpg]

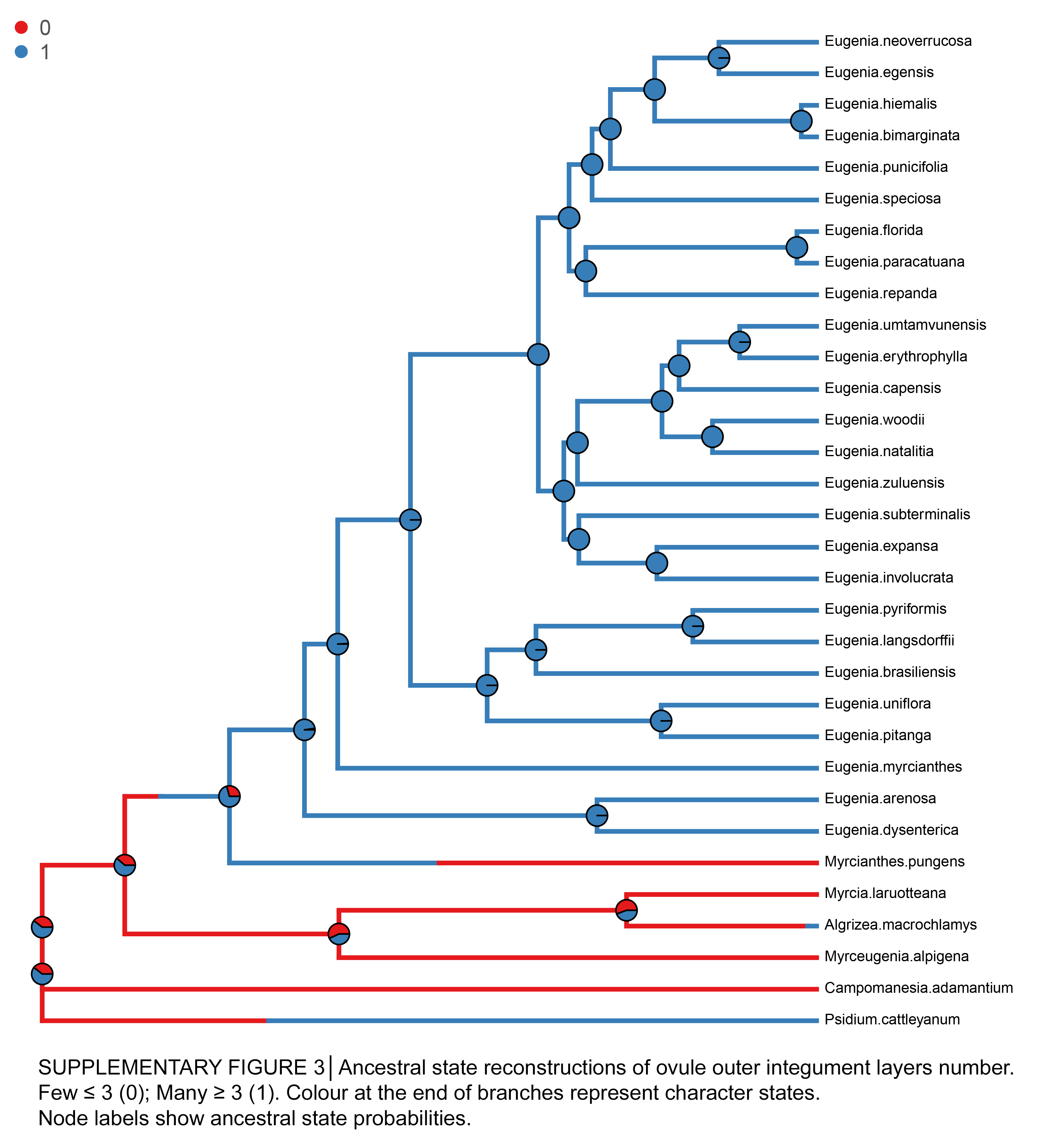

Supplement: Supplementary file 6 [file Image_3.jpg]

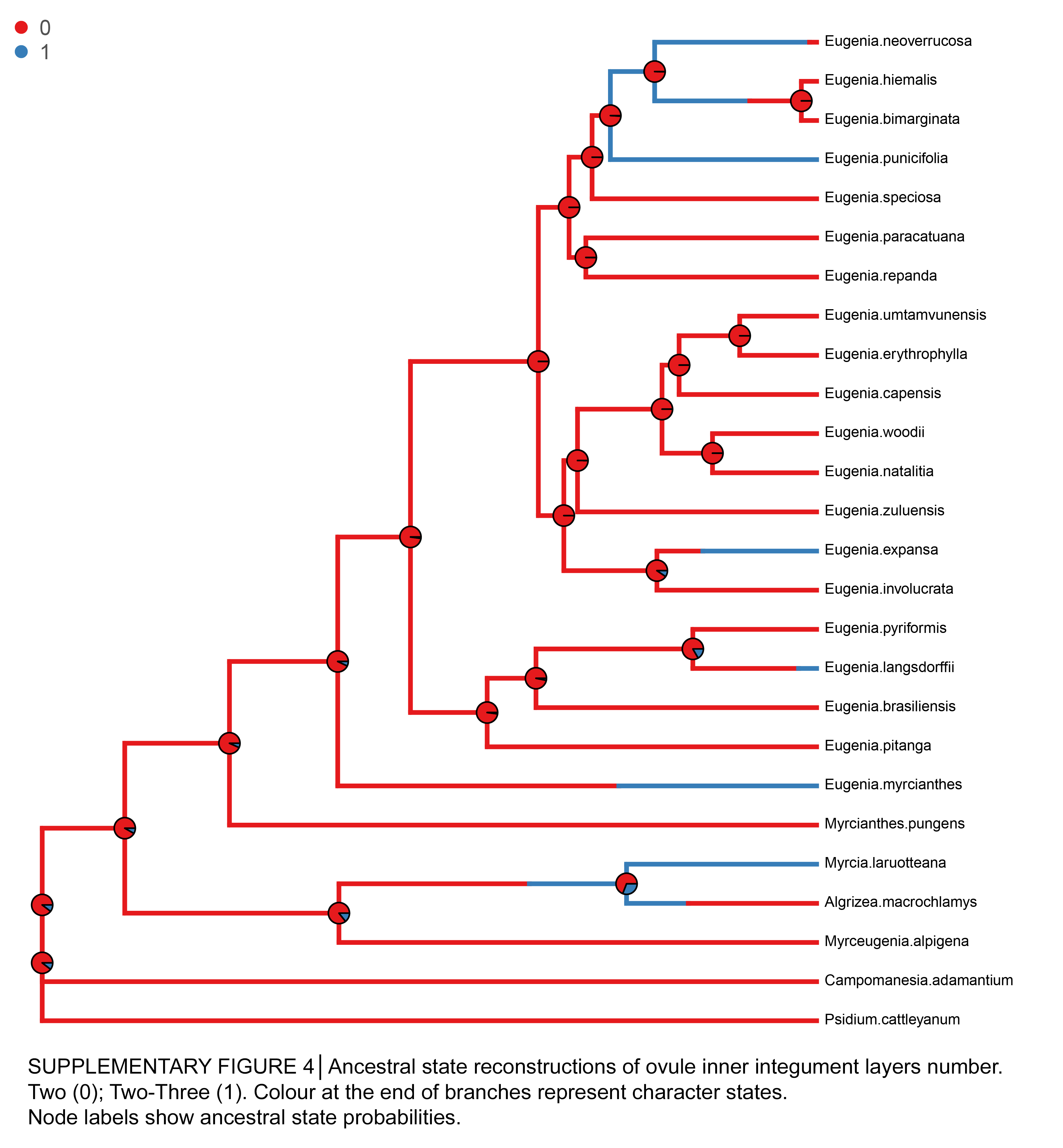

Supplement: Supplementary file 7 [file Image_4.jpg]

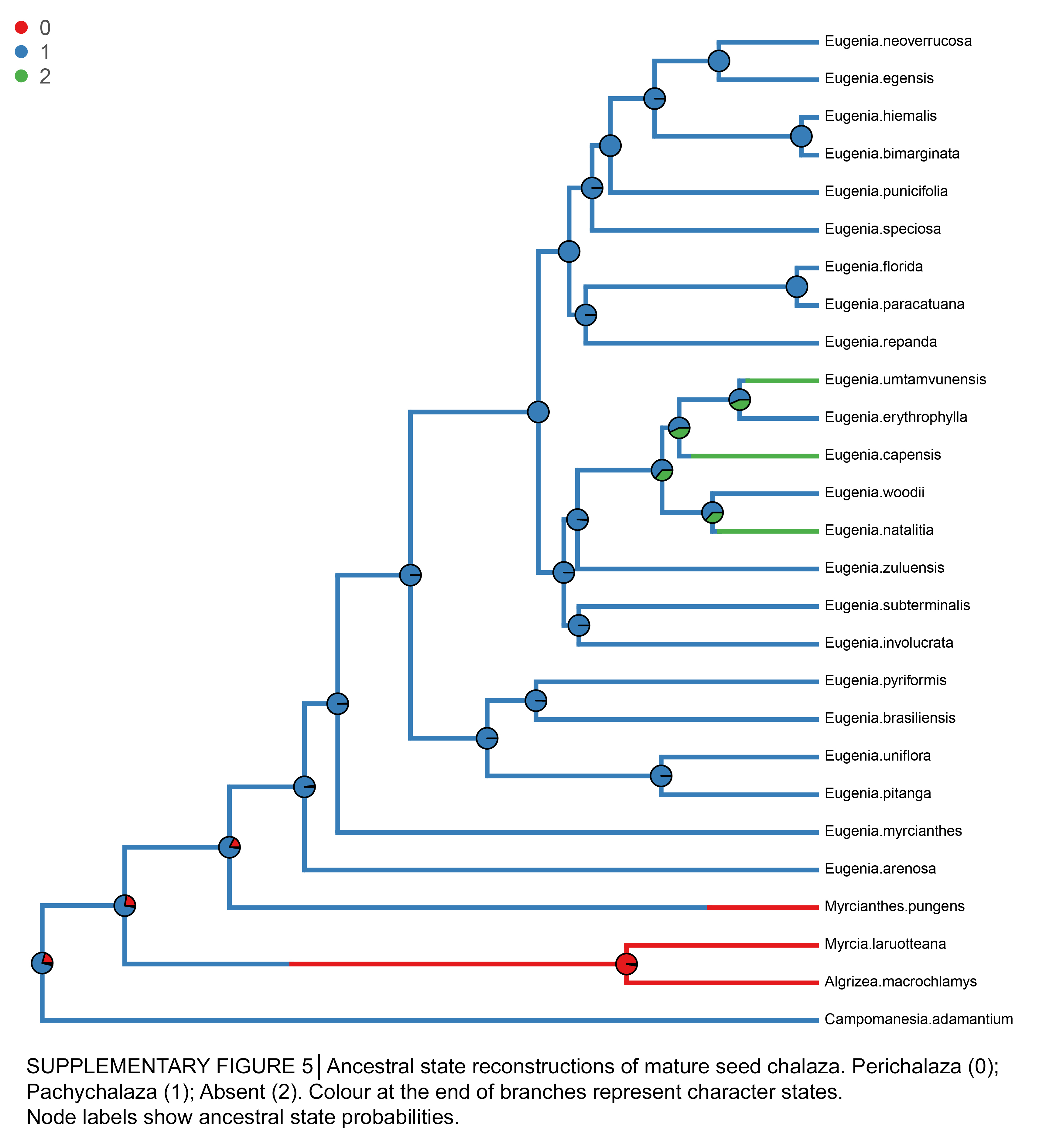

Supplement: Supplementary file 8 [file Image_5.jpg]

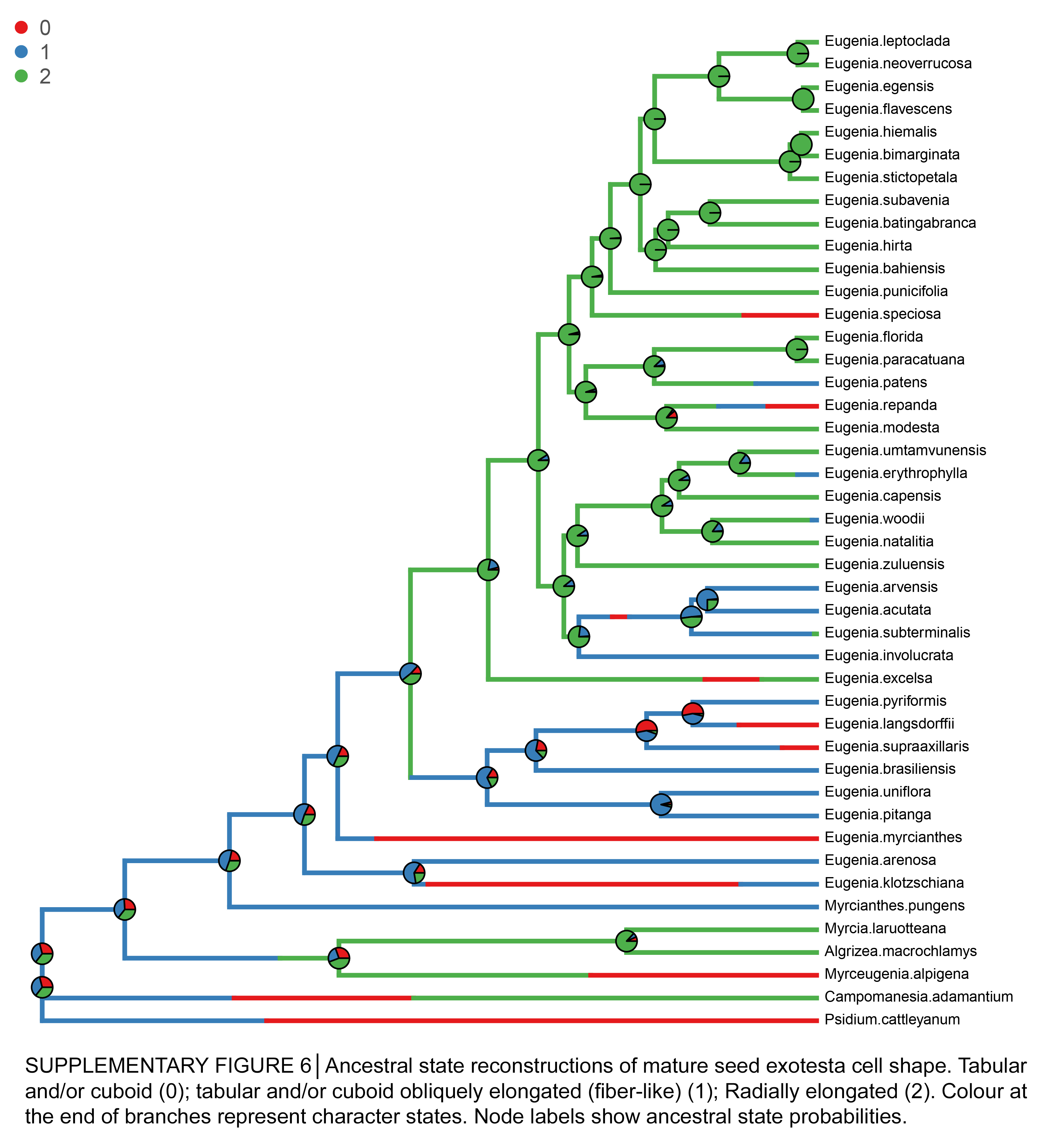

Supplement: Supplementary file 9 [file Image_6.jpg]

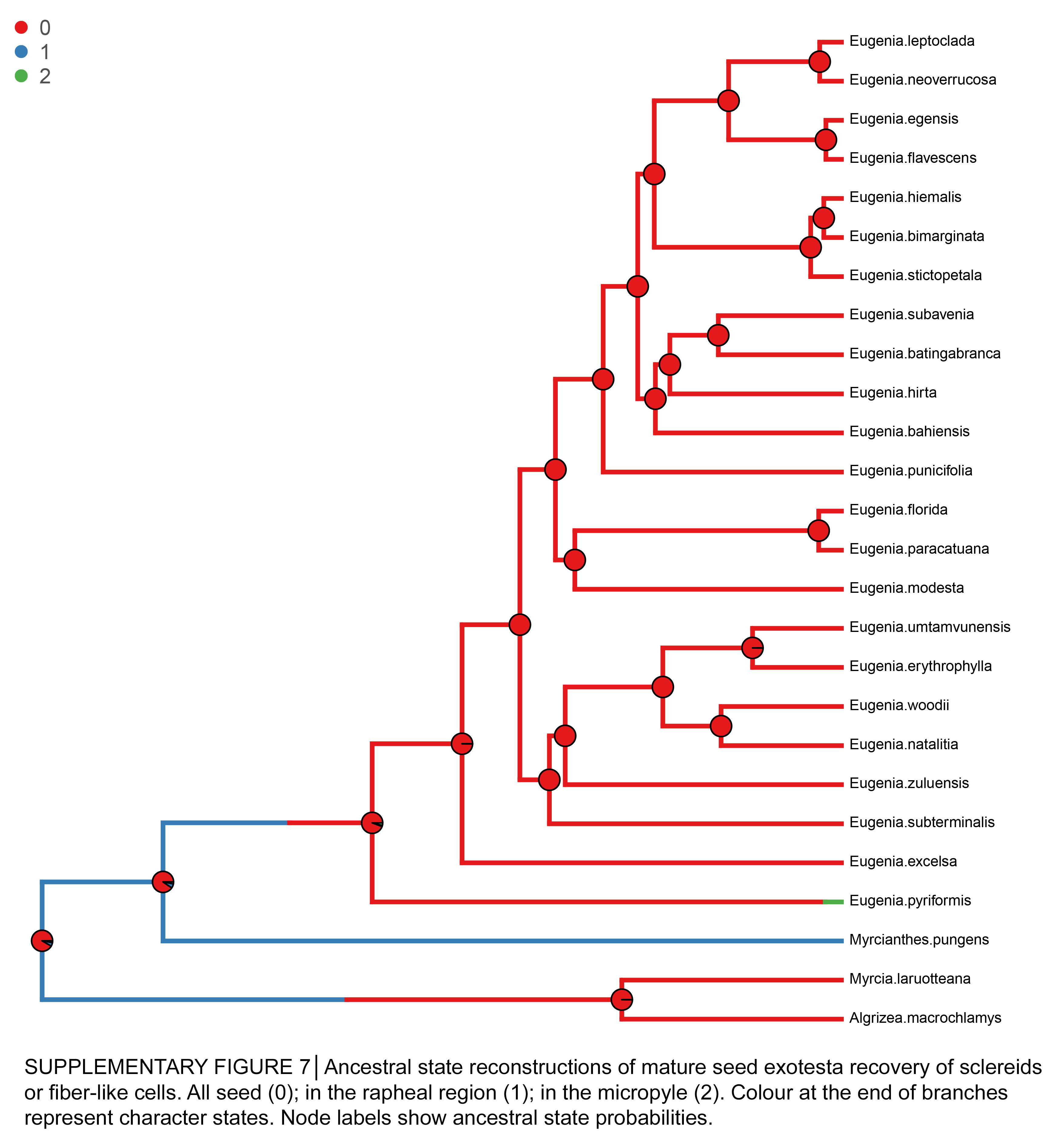

Supplement: Supplementary file 10 [file Image_7.jpg]

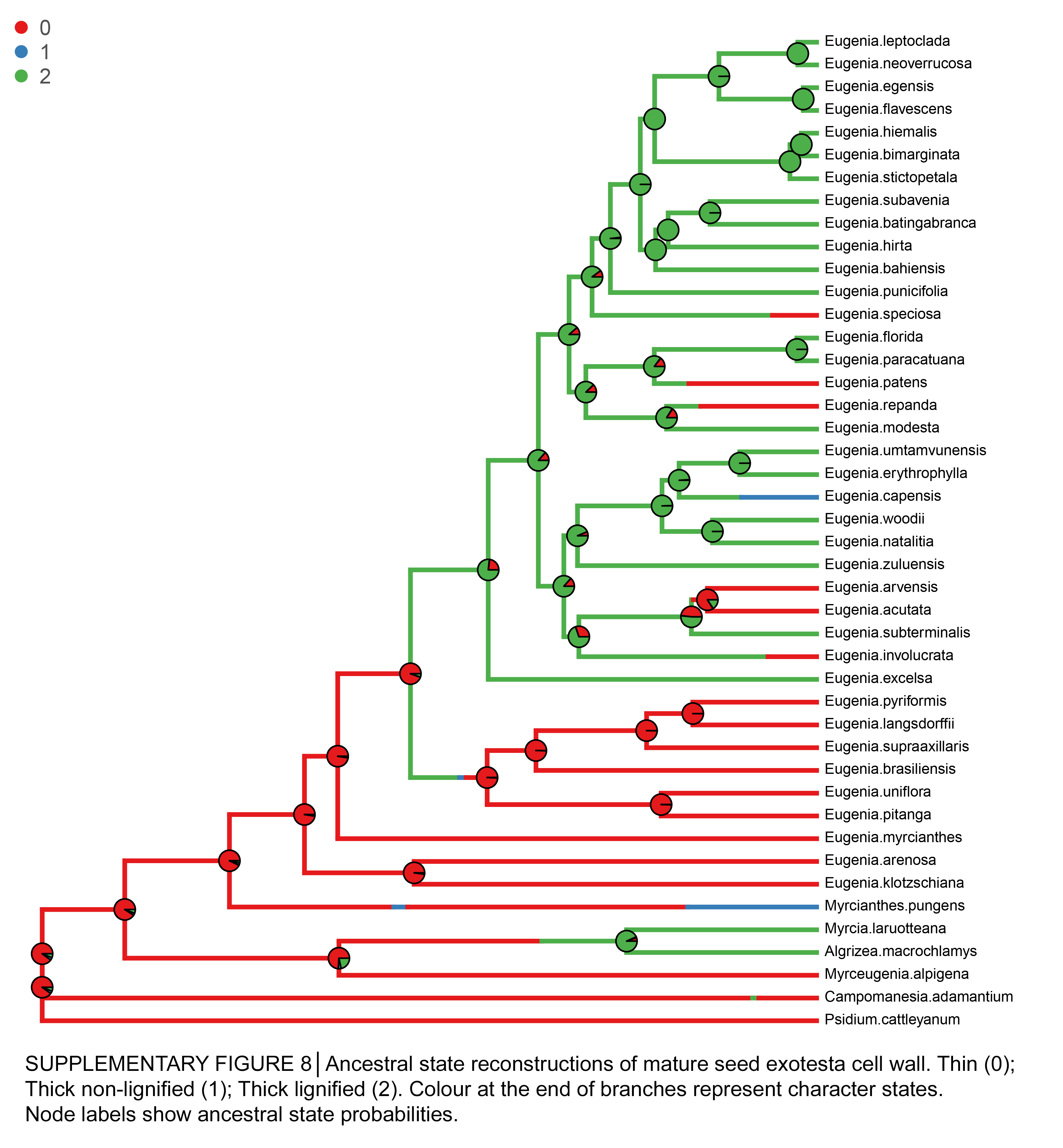

Supplement: Supplementary file 11 [file Image_8.jpg]

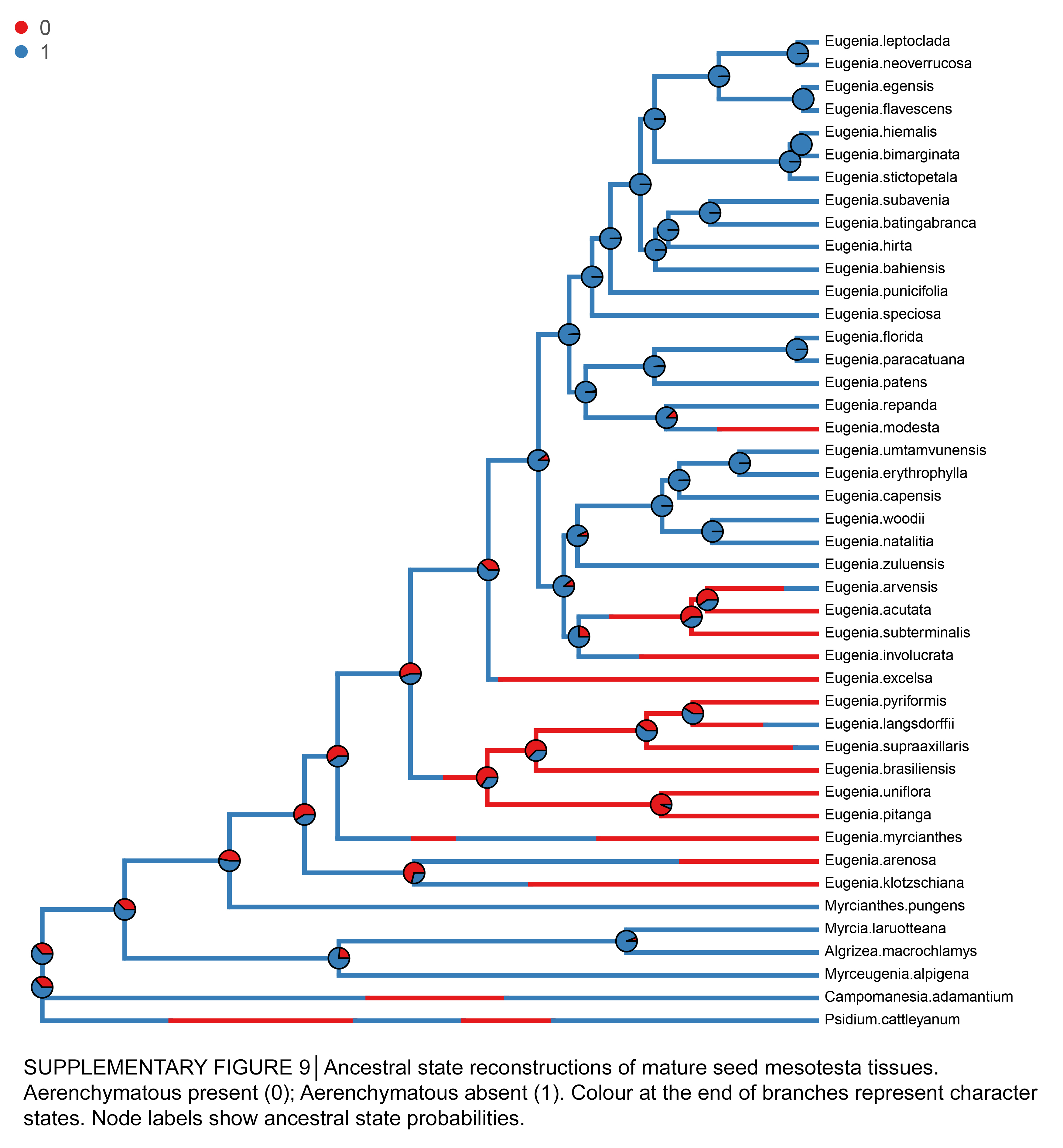

Supplement: Supplementary file 12 [file Image_9.jpg]

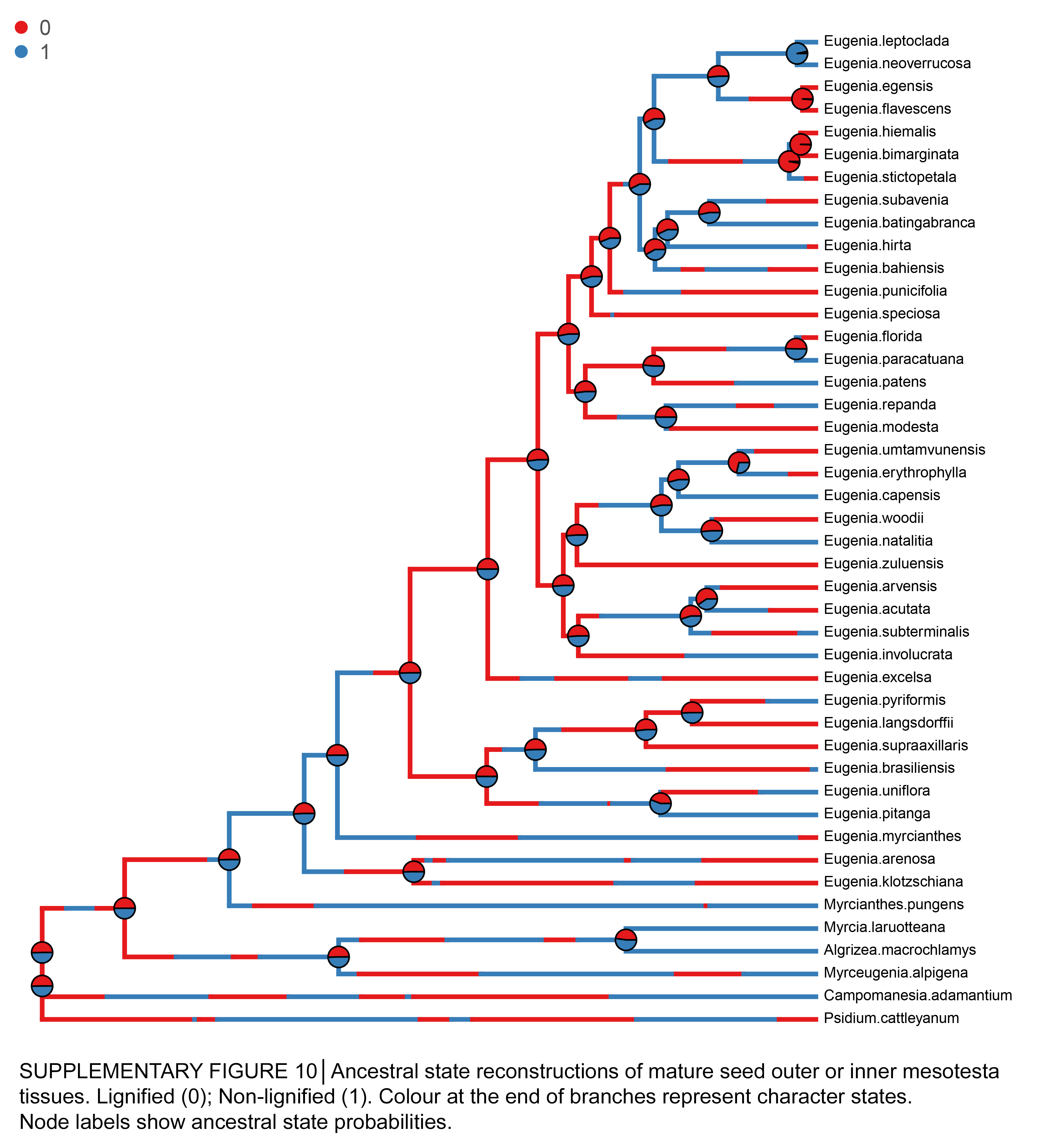

Supplement: Supplementary file 13 [file Image_10.jpg]

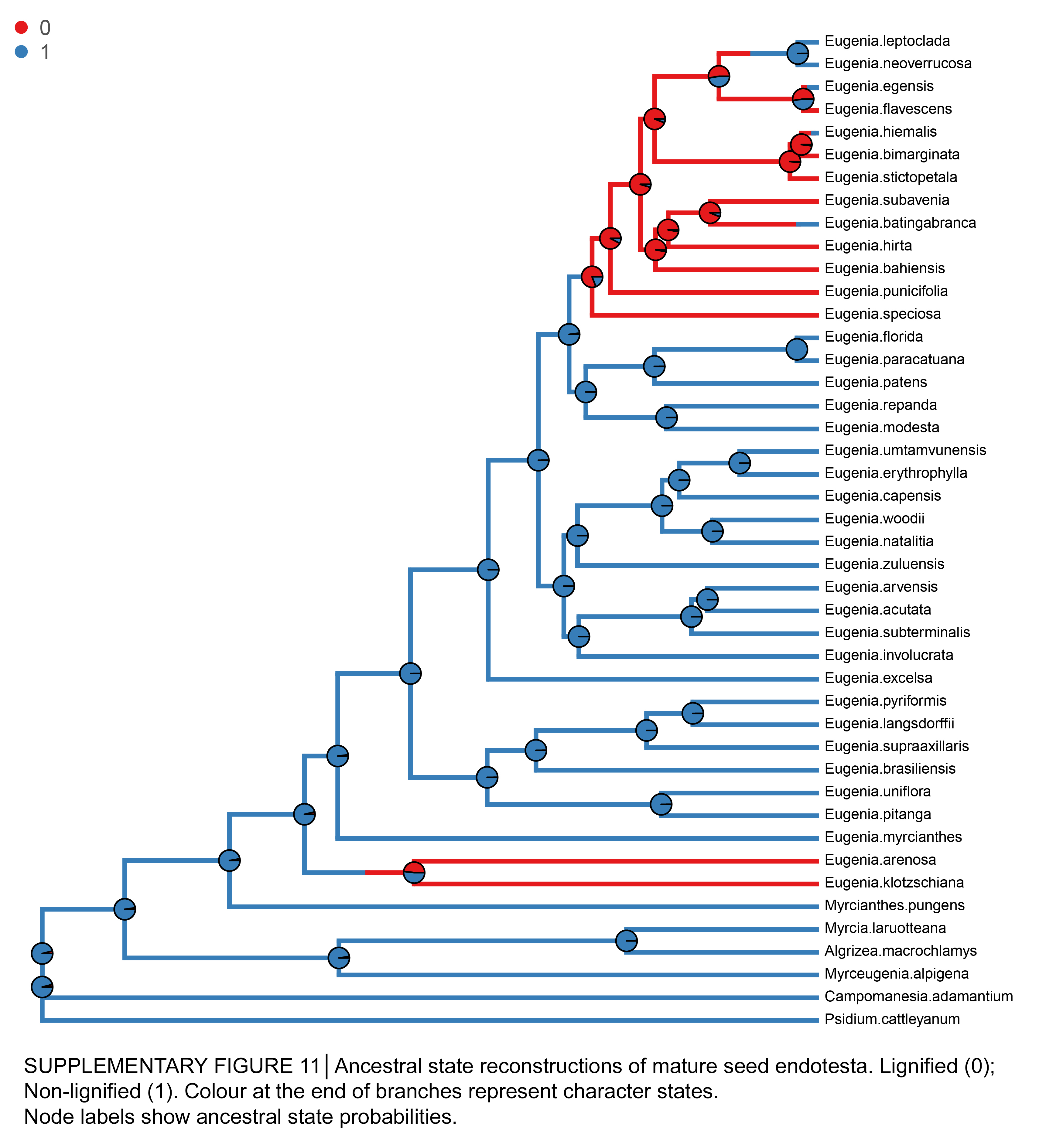

Supplement: Supplementary file 14 [file Image_11.jpg]

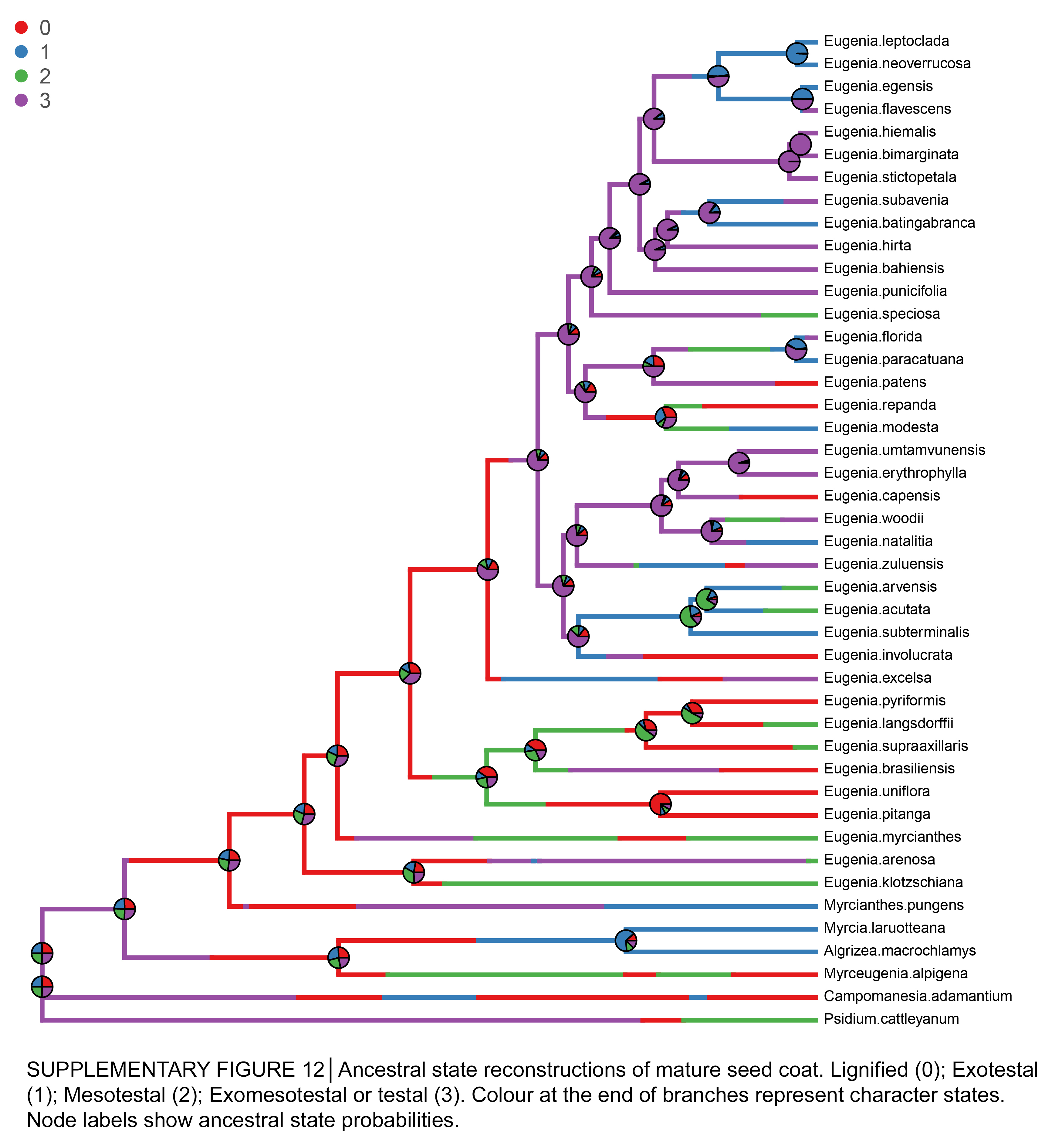

Supplement: Supplementary file 15 [file Image_12.jpg]
